# Organ-specific characteristics govern the relationship between histone code dynamics and transcriptional reprogramming during nitrogen response in tomato

**DOI:** 10.1038/s42003-023-05601-8

**Published:** 2023-12-04

**Authors:** Russell Julian, Ryan M. Patrick, Ying Li

**Affiliations:** 1 Department of Horticulture & Landscape Architecture, Purdue University, West Lafayette, IN 47907 USA; 2 Center for Plant Biology, Purdue University, West Lafayette, IN 47907 USA

**Keywords:** Plant molecular biology, Epigenetics, Machine learning

## Abstract

Environmental stimuli trigger rapid transcriptional reprogramming of gene networks. These responses occur in the context of the local chromatin landscape, but the contribution of organ-specific dynamic chromatin modifications in responses to external signals remains largely unexplored. We treated tomato seedlings with a supply of nitrate and measured the genome-wide changes of four histone marks, the permissive marks H3K27ac, H3K4me3, and H3K36me3 and repressive mark H3K27me3, in shoots and roots separately, as well as H3K9me2 in shoots. Dynamic and organ-specific histone acetylation and methylation were observed at functionally relevant gene loci. Integration of transcriptomic and epigenomic datasets generated from the same organ revealed largely syngenetic relations between changes in transcript levels and histone modifications, with the exception of H3K27me3 in shoots, where an increased level of this repressive mark is observed at genes activated by nitrate. Application of a machine learning approach revealed organ-specific rules regarding the importance of individual histone marks, as H3K36me3 is the most successful mark in predicting gene regulation events in shoots, while H3K4me3 is the strongest individual predictor in roots. Our integrated study substantiates a view that during plant environmental responses, the relationships between histone code dynamics and gene regulation are highly dependent on organ-specific contexts.

## Abstract (web summary)

Analysis of ChIP-Seq of multiple histone modifications across shoots and roots in two nutrient supply conditions, coupled with machine learning, reveals new organ-specific patterns of epigenetic regulation in response to nitrate in tomatoes.

## Introduction

Nitrogen (N) is an essential plant macronutrient and a limiting factor for plant growth^1,2^. In modern agriculture, N is often amended in soil by applying chemical N fertilizer, which effectively boosts crop yield but contributes to environmental problems and climate change, impacting biodiversity and human health^3,4^. A comprehensive understanding of the molecular mechanisms of N uptake and assimilation is desired to improve nitrogen use efficiency and agricultural sustainability. In aerobic soil in a temperate climate, N is primarily taken up from the soil as nitrate through nitrate transporters (NRTs) in the roots encoded by the *NITRATE TRANSPORTER 1/PEPTIDE TRANSPORTER* *Family (NPF)*^1,5–9^. Nitrate is then assimilated in the roots or transported via NPF transporters to the shoots for assimilation^5^. During N assimilation, nitrate is first reduced to nitrite by nitrate reductase (encoded by *NIAs*), and then reduced to ammonium by nitrite reductase (encoded by *NIRs*)^1^. Ammonium, absorbed directly from the soil through ammonium transporters (AMTs) or produced from nitrate, is assimilated through the glutamine synthetase (GS)/glutamine-2-oxoglutarate aminotransferase (GOGAT) cycle^10^ into the amino acids glutamate and glutamine. The amino groups of glutamate and glutamine can then be transferred between carbon skeletons to produce other amino acids^11^ via aminotransferases e.g., asparagine synthetase (ASN)^12^.

Nitrate is not only a macronutrient but also a signaling molecule. Nitrate is sensed by the transceptor NPF6.3, a dual-function transporter and sensor protein^13,14^, triggering a signaling cascade and widespread reprogramming of gene expression on a scale of minutes to hours^15,16^ that results in physiological and developmental changes within days, including enhanced chlorophyll biosynthesis in the shoots and morphological changes in the roots^17,18^. NLP7, a member of the NIN-like protein family (NLPs), is a master regulator which translocates into the nucleus within minutes of nitrate supply to bind and regulate hundreds of nitrate-responsive genes^19^ and has also recently been recognized to function as a nitrate sensor protein^20^. Many other transcription factors (TFs) involved in nitrate responses have been characterized^5^: *HYPERSENSITIVE TO LOW PI-ELICITED PRIMARY ROOT SHORTENING 1 (HRS1)* and other members in the *HRS1* *HOMOLOG* *FAMILY* (*HHO*) are induced by a supply of nitrate within 10 min to regulate nutrient metabolism and root growth^21^; Basic leucine zipper 1 (bZIP1), another master regulator of N responses, acts through a hit-and-run mechanism to activate a large set of genes in response to N supply^22^; WRKY1 integrates light response and N signaling to coordinate carbon and N metabolism^23^; *LATERAL BOUNDARY DOMAIN CONTAINING PROTEIN 37 (LBD37)*, *LBD38*, and *LBD39* are induced by N and function as repressors of *NPF6.3*, *NRT2.1* and *NIA1* to fine-tune nitrate response^24^.

Regulation of gene networks by TFs occurs in the context of the local chromatin landscape. At the most basic level, this consists of genomic DNA wrapping around histone octamers to form nucleosomes^25^. Posttranslational modifications of histone proteins at their N-terminal tails are known to affect chromatin organization with resultant effects on gene expression^26^. The combination of a wide range of possible histone modifications, such as methylation and acetylation at different amino acid positions on histone tails, forms the histone code to extend the information potential of the genomic DNA at a given gene locus^27^. Histone acetylation reduces the positive charge of histones to create an open chromatin state, which is generally associated with active gene transcription^28^. Histone methylation can be associated with gene activation or repression depending on the amino acid substrates: histone subunit 3 lysine (K) 4 trimethylation (H3K4me3) and H3K36me3 are usually associated with active gene expression, while H3K27me3 and H3K9me2 are considered repressive histone marks associated with gene silencing^29,30^. Histone methylation and acetylation are reversible marks maintained by the coordinated function of writer and eraser enzymes. Histone acetylation is deposited by the histone acetyltransferase (HAT) writer proteins and erased by histone deacetylases (HDACs)^31^. Similarly, histone methylation is written by SET DOMAIN GROUP (SDG) proteins and erased by demethylases such as Jumonji (JMJs)^32^.

In plants, histone modifications play a key role in interfacing external signals and cellular gene expression to potentiate adequate physiological responses to the environment. For instance, histone deacetylases HDA6^33,34^, HDA9^35^, HDA19^36^, and HDT2^37^ are involved in plant response to abiotic or biotic stresses. H3K4 demethylases JMJ16 and JMJ17 mediate drought response^38^. H3K36me3, partially mediated by SDG8, was shown to regulate response to light^39^, pathogen^40^, and temperature^41^. Recently, a few studies have suggested that chromatin regulation plays an active role in plant nutrient responses, especially responses to N. HIGH NITROGEN INSENSITIVE 9 (HNI9), a component of the Pol II complex, was shown to repress *NRT2.1* during high N supply through increased H3K27me3^42^. H3K27me3 was also shown to be deposited at *NRT2.1* locus even when *NRT2.1* is highly induced, possibly to attenuate its expression^43^. In maize, the chromatin remodeling protein ZmCHB101 was found to contribute to nitrate response in part by regulating H3K4me3 and H3K27me3 dynamics at the *NRT2.1* and *NRT2.2* genes^44^. Levels of H3K9ac and H3K27ac at autophagy-related genes were observed to be dynamically regulated by HDA9 in response to nitrate starvation^45^. A genome-wide study found that SDG8 affects H3K36me3 levels and RNA processing in response to nitrate treatment^46^; however, this study focused on aerial tissues, while the epigenomic changes in roots, the organ that first senses and uptakes N, remain unknown. In fact, although it has been well documented that chromatin modifications are dependent on organ or tissue context^47^, it has remained unclear whether the same external stimulus triggers similar or distinct chromatin changes in different organs such as shoots *vs* roots. In addition, while previous studies typically focused on either specific genes, gene families, or pathways, and assayed a limited number of histone marks, an in-depth understanding of the histone code requires multiple histone marks to be evaluated simultaneously genome-wide to elucidate the individual and combinatorial effects of histone modifications on gene expression. Finally, to date, most studies have been performed in Arabidopsis, while knowledge on epigenetic regulation in crop species is relatively scarce.

Here, we present an extensive genome-wide study of five histone marks: H3K4me3, H3K27ac, H3K36me3, and H3K27me3, in two organs, shoots and roots, and additionally H3K9me2 in shoots, in response to the nutrient nitrate in tomato plants. The marks were chosen based on previous evidence for their involvement in nitrate-responsive gene regulation^42–46^ and the availability of reliable and high-quality antibodies for chromatin immunoprecipitation sequencing (ChIP-Seq). Our results suggest that histone modifications are responsive to the N supply in an organ-specific manner, with H3K27ac being the most dynamic. Overlaying transcriptome data collected from the same organs, we found that while permissive marks are often associated with gene activation, and vice versa, a non-canonical pattern of increasing H3K27me3 was observed at a group of up-regulated genes in the shoots. Finally, we applied machine learning approaches to understand the contribution of histone marks, individually or in combination, in predicting gene regulation. While combinatorial information from multiple histone marks greatly increased accuracy of prediction of gene regulation, accuracy of those predictions was mostly dependent on H3K36me3 in the shoots and H3K4me3 in the roots.

## Results

### A supply of nitrate triggers organ-specific changes of histone modifications at specific gene loci

**Fig. 1:**
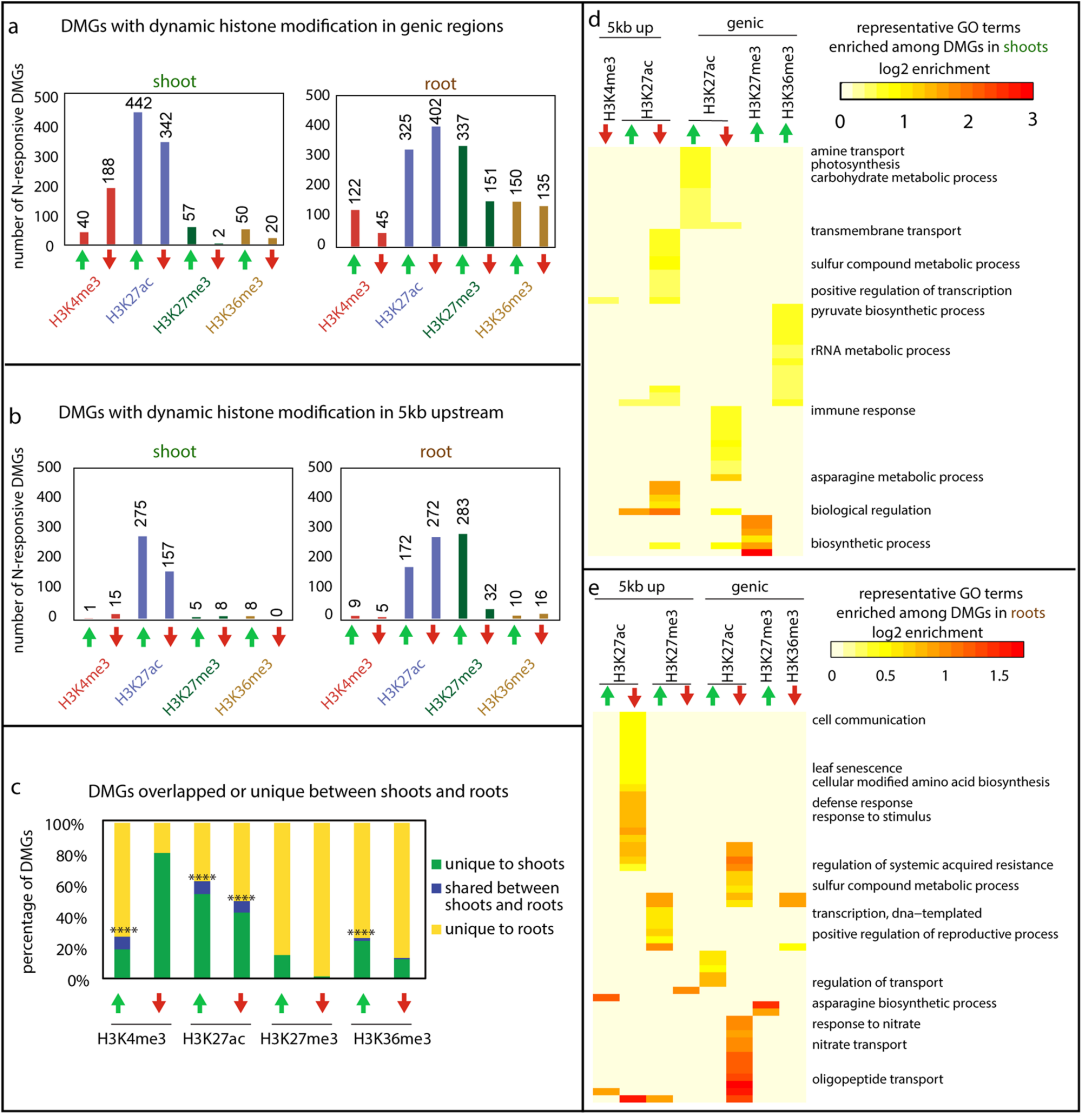
Nitrogen treatment triggered organ-specific dynamic changes in various histone modifications at gene loci involved in distinct biological processes. **a** Number of differentially modified genes (DMGs) with significantly different levels of H3K4me3, H3K27ac, H3K27me3, or H3K36me3 in the transcribed regions of genes (i.e., genic region) in the +N treated samples compared to the −N controls. **b** Number of DMGs with significantly different levels of histone modifications in the 5 kb upstream of TSS (i.e., promoter) in the +N treated samples compared to the −N controls. **c** The percentage of DMGs with dynamic genic marks that are shared between shoots and roots (blue columns), unique to the shoots (green columns), or unique to the roots (yellow columns). The significance of the overlap between shoots and roots was determined using hypergeometric distribution against a whole genome background, and a significant overlap is indicated by asterisks: (•*p* < 0.1; *p < 0.05; **p < 0.01; ***p < 0.001; ****p < 0.0001). **d**, **e** Heatmap depicting the fold enrichment of signaling GO terms significantly enriched among the DMGs identified in shoots (**d**) or in roots (**e**). Significantly over-represented GO terms among individual groups of DMGs were first determined using ShinyGO (enrichment FDR < 0.05). Semantic redundancy within a GO term list comprising all terms significantly over-represented in at least one set of DMGs was determined by ReviGO^98^. GO terms with a dispensability score >0.5 in roots or >0.4 in shoots were removed to only keep representative GO terms. A selection of GO terms are labeled in the figure, while the full list of GO terms are in Supplementary Data 7. The log2 value of enrichment level (representation of a GO term in the group of DMGs *vs* the representation of this GO term in whole genome background) is shown, and the value is set to 0 if the GO term does not pass the statistical cutoff in the corresponding gene sets.

To investigate the organ specificity of dynamic histone modifications in response to N changes, we treated 3-week-old tomato seedlings (*Solanum lycopersicum*, cultivar M82) with four days of N starvation, followed by N-supply (2.8 mM NO_3_^−^; +N) or continued N-starvation (2.8 mM Cl^−^ as a control; −N) treatments (Supplementary Fig. 1). Growth in +N conditions led to increased biomass, greater shoot-to-root ratio, and higher chlorophyll content compared to the −N controls after seven days (Supplementary Fig. 2). To investigate the chromatin regulatory mechanisms underlying response to N, we assayed the genome-wide profiles of four histone modifications, H3K4me3, H3K27ac, H3K27me3, and H3K36me3, by ChIP-Seq in shoots and roots six hours following +N or −N treatment (Supplementary Fig. 1). One additional histone modification, H3K9me2, was assayed for shoots only, as described in the following section. The six-hour time point was chosen to capture the relatively early regulatory events that would lead to physiological divergence, while allowing enough time for the histone modifications to respond to the N signal^48^. The analyses of our ChIP-Seq data uncovered hundreds to thousands of dynamic islands, i.e., genomic regions associated with H3K4me3, H3K27ac, H3K27me3, or H3K36me3 modifications that are significantly different between +N and −N conditions (FDR < 0.05 and fold-change > 1.5) (Supplementary Fig. 3a, b). The majority of dynamic H3K4me3 and H3K36me3 islands are colocalized with the transcribed region of annotated genes (i.e., genic region; Supplementary Fig. 3c, d). By contrast, the genomic islands with dynamic H3K27ac or H3K27me3 are not only associated with genic regions, but also located to putative promoters (i.e., 5 kb upstream of the start of annotated genes), and located in intergenic regions (>5 kb from any gene) (Supplementary Fig. 3c, d). Differentially modified genes (DMGs) associated with dynamic histone modifications were identified, both in the genic region (Fig. 1a) and in the putative upstream promoters (Fig. 1b) for roots or shoots separately (Supplementary Data 1). Among the four histone marks assayed, H3K27ac appeared to be the most strongly responsive to N supply; dynamic H3K27ac occurs at hundreds of gene loci, while dynamic H3K27me3, H3K4me3, and H3K36me3 are observed at a more limited set of gene loci (Fig. 1a, b). Moreover, different histone marks seem to be regulated at gene loci involved in distinct biological processes (Supplementary Data 7; Fig. 1d, e): for example, in the shoots, genic H3K27ac is increased by N supply at genes involved in photosynthesis and carbon metabolism, while genic H3K36me3 is increased at gene loci involved in rRNA metabolism (Fig. 1d), despite both marks having an accepted role associated with gene activation.

We next asked whether these dynamic histone modification changes are specific to one organ or shared between shoots and roots. In general, the majority of DMGs with dynamic *genic* histone modification (>91%) are organ specific, in that they are significantly responsive to N supply only in shoots or in roots (Fig. 1c). The comparison of DMGs with dynamic histone modification in the promoter was less meaningful due to the limited number of significant genes observed. Interestingly, we observed varying levels of organ specificity for different histone modifications. Dynamic change of H3K27me3 is highly specific to roots, as hundreds of DMGs *hyper-* or *hypo-*methylated with H3K27me3 are observed in the roots but a much more limited number of DMGs are detected in the shoots (Fig. 1a). Indeed, there was no overlap between the H3K27me3 DMGs in shoots and those in roots (Fig. 1c). Similarly, the changes of H3K36me3 were also more prominent in the roots than in the shoots (Fig. 1a). Genic H3K4me3 was more likely to increase than decrease in roots in response to N supply while in shoots the opposite pattern was observed (Fig. 1a). Finally, H3K27ac is the least organ-specific of the four marks, in that it is dynamic at hundreds of gene loci in both organs (Fig. 1a) with a significant portion of overlapped DMGs observed in both shoots and roots (Fig. 1c).

In summary, we observed dynamic and organ-specific changes of histone marks in response to changing N supply occurring at distinct functionally relevant gene loci for different marks. Generally, these events were limited to either shoots or roots and the level of response for different marks varied greatly. Overall, our epigenomic data uncovered notably different chromatin dynamics between the two organs in response to N supply, indicating that distinct epigenetic machinery operates downstream of nitrate signaling pathways to modify chromatin at specific target genes and manifest proper response in each organ.

### Integrating epigenome and transcriptome data provides insight into the regulation of N regulatory and metabolic genes

To understand how the observed epigenomic changes affect gene expression, we profiled and analyzed the shoot and root transcriptome of tomato plants treated with −N and +N conditions from tissues harvested in parallel with the ChIP-Seq samples (Supplementary Fig. 1). In total, we identified 1331 DEGs that are up-regulated and 1304 down-regulated by a supply of N in roots, and 2760 up-regulated and 1534 down-regulated DEGs in shoots (Supplementary Data 2), using DEseq2. Concordant with the observed organ-specificity of DMGs, the majority of DEGs were also organ-specific in that 85% of up- and down-regulated DEGs changed significantly in only one organ.

In the roots, up-regulated DEGs are enriched with functional annotations for transmembrane transport (nitrate transport), ribosome biogenesis, and negative regulation of peptidase activity, indicating activation of N uptake and protein synthesis pathways in response to increasing N supply (Supplementary Data 3a). These include nitrate transporter *NRTs* (*Solyc11g069760.1*, *Solyc08g007430.2*, *Solyc05g006990.3*, and *Solyc11g069735.1*), as well as genes encoding enzymes for N assimilation and amino acid biosynthesis such as *NIA* (*Solyc11g013810.3*), *NIR* (*Solyc01g108630.3*, *Solyc10g050890.2*), and *GS* (*Solyc04g014510.3*) (Supplementary Data 2). Interestingly, down-regulated DEGs are also enriched with the biological process ‘transport’ (Supplementary Data 3b), including transporters for other mineral nutrients such as manganese (*Solyc01g095510.3*), magnesium (*Solyc05g012220.3*), calcium (*Solyc07g006370.1*), and zinc (*Solyc07g043200.2*, *Solyc07g043230.3*) (Supplementary Data 2). Therefore, the diverse mineral nutrient uptake and transport processes in the roots seem to adjust in response to the supply of N. In the shoots, the up-regulated genes are enriched with GO terms ‘translation’, ‘chlorophyll biosynthetic process’, and ‘nitrogen compound transport’ (Supplementary Data 3c), including genes encoding N transporters and N assimilation enzymes such as *NRT* (*Solyc06g074990.3* and *Solyc05g006990.3*), *NIA* (Solyc11g013810.3), *NIR (Solyc01g108630.3* and *Solyc10g050890.2*), *GS* (*Solyc01g080280.3* and *Solyc04g014510.3*), and *ASN* (*Solyc04g055200.3*) (Supplementary Data 2). The down-regulated DEGs in shoots are enriched with regulatory processes and signaling pathways, as well as aging and leaf senescence (Supplementary Data 3d). Overall, our transcriptome analyses suggested that N assimilation and growth processes are activated and signaling cascades are reprogrammed in response to N supply in the shoots.

**Fig. 2:**
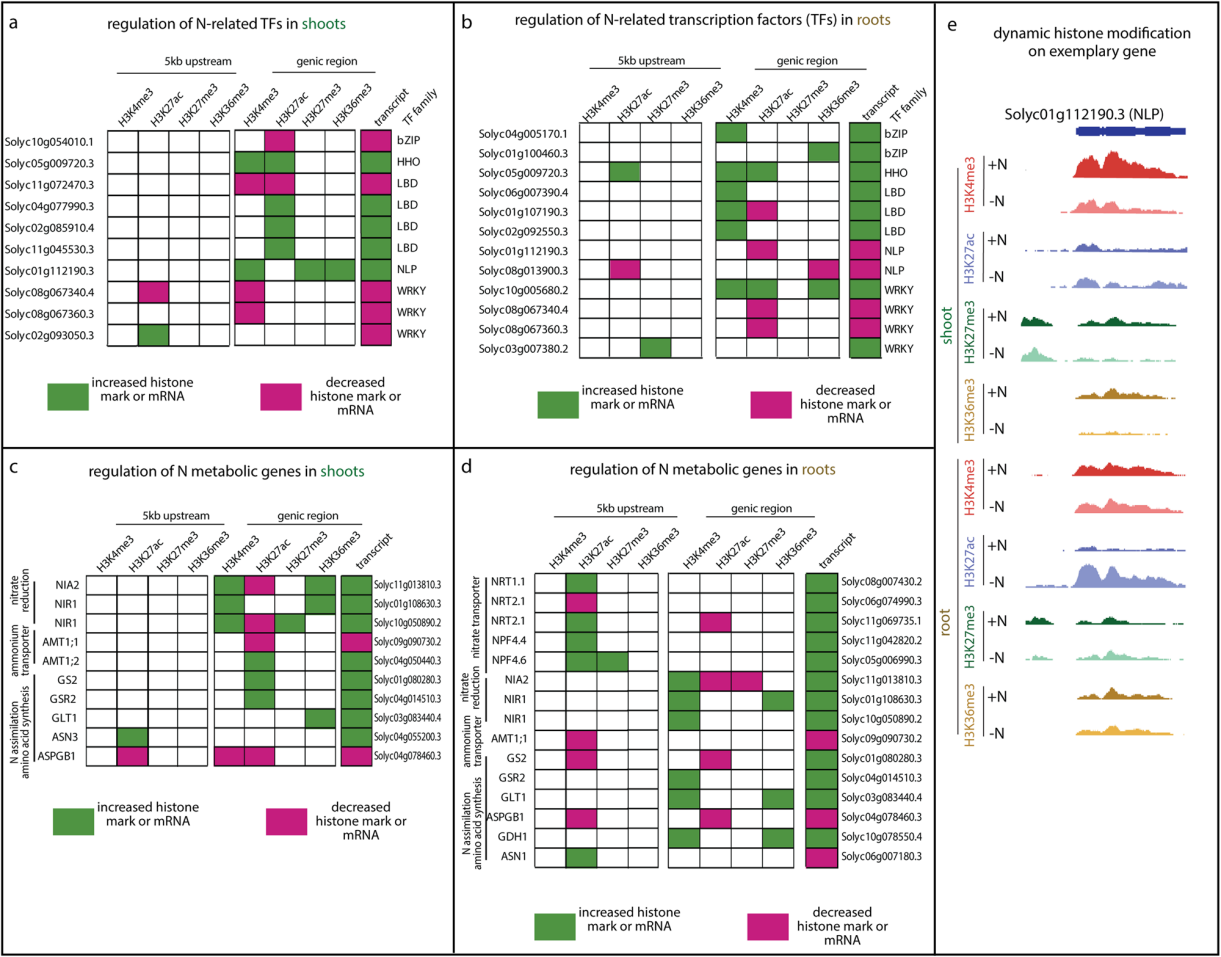
Nitrogen treatment causes complex changes in chromatin modifications and transcript levels of genes encoding known TF families, enzymes, and transporters involved in nitrogen responses. Genes encoding TF families (**a**, **b**) or enzymes and transporters (**c**, **d**) are shown with their significant changes in histone modifications and transcript levels. Green color indicates up-regulation of mRNA level or increased level of histone modification, while magenta color indicates down-regulation of mRNA levels or decreased histone modifications. **e** Histone ChIP-Seq coverage showing dynamic histone modifications along gene body of an *NLP* TF (*Solyc01g112190.3*) between −N and +N conditions. The sequencing depth was scaled to library size. Representative results from one of three independent biological replicates are shown.

Overlaying epigenomic and transcriptomic data generated from the same samples provided insight to the complexity of chromatin modification at responsive regulatory and metabolic genes essential for N metabolism. TF families such as bZIPs^22^, HHOs^49^, LBDs^24^, NLPs^19^, and WRKYs^23^ are known to be involved in regulating N responses. Our results showed that members of these TF families are indeed regulated by N at both epigenetic and transcriptional levels (Fig. 2a, b), with many marked by complex histone codes that are specific to the individual gene locus and organ context. For example, *Solyc01g112190.3*, encoding an NLP family TF with similarity to the master regulator NLP7^19^, shows organ-specific regulatory changes, being up-regulated in shoots but down-regulated in roots, with distinct epigenetic dynamics in the two organs. The gene is associated with increased H3K4me3, H3K27me3, and H3K36me3 in shoots, but in roots decreasing genic H3K27ac is observed (Fig. 2a, b, e). The different epigenetic states could possibly mediate the organ-specific transcriptional regulation of this *NLP*. We also investigated the epigenetic and transcriptional regulation patterns of genes central to N transport and metabolism^22^ (Fig. 2c, d). In roots, multiple genes encoding nitrate transporter family proteins (NRTs and NPFs) are up-regulated and associated with dynamic H3K27ac in the promoter (Fig. 2d), a pattern not observed in the shoots (Fig. 2c). By contrast, in both organs genes involved in nitrate reduction, ammonium transport, and N assimilation and amino acid biosynthesis are regulated at epigenetic and transcriptional levels. Specifically, genes encoding essential enzymes in nitrate reduction and assimilation, such as nitrate reductase (*Solyc11g013810.3*), nitrite reductase (*Solyc01g108630.3* and *Solyc10g050890.2*), glutamine synthetase (*Solyc01g080280.3* and *Solyc04g014510.3*), and glutamate synthase (*Solyc03g083440.4*) are up-regulated in both shoots and roots (Fig. 2c, d). Some of these genes share dynamic histone modification regulations between the two organs; for example, *NIA* and *NIR* genes displayed increased H3K4me3 in both shoots and roots (Fig. 2c, d). Other genes, such as *GS* (*Solyc01g080280.3*), displayed discrete epigenetic modifications in shoots *vs* roots despite being transcriptionally up-regulated in both organs (Fig. 2c, d). These observations add complexity to traditional views of gene regulation, revealing that seemingly similar up-regulated genes can be under different chromatin regulatory mechanism in different organs, possibly affecting the duration, magnitude, memory, or transcriptional processing of these gene activation events.

### Global relationship between epigenome and transcriptome responses

**Fig. 3:**
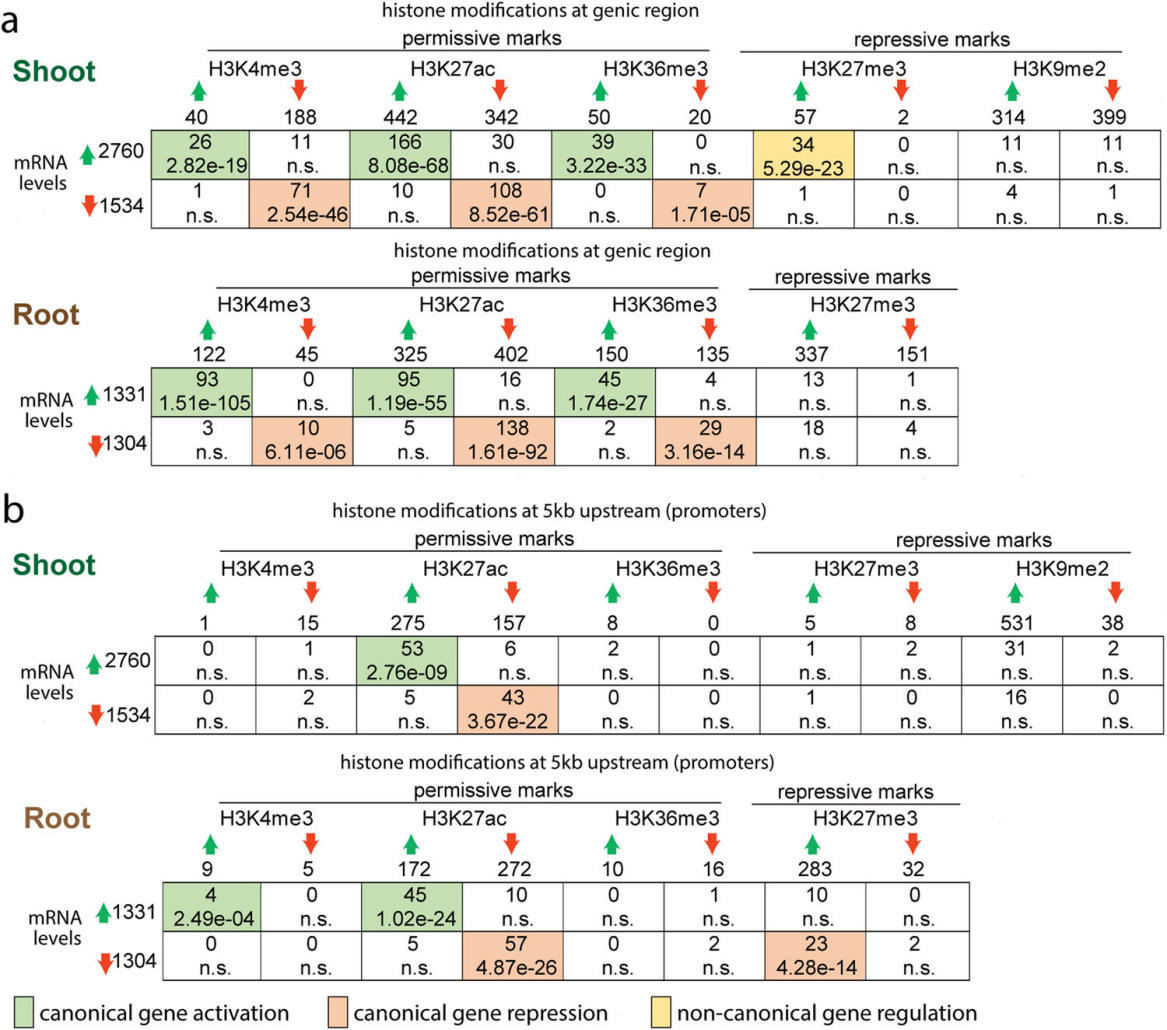
Overlapping differentially modified genes (DMGs) with differentially expressed genes (DEGs) uncovered canonical and non-canonical gene regulatory patterns. The rows represent the number of DEGs up-regulated or down-regulated at the mRNA level in +N samples compared to −N controls in shoots or roots. The columns represent the number of DMGs with increased or decreased histone modifications between +N and −N samples, in shoots or roots, separately, for genic regions (**a**) or promoters (**b**). Each cell represents the overlap between the DMGs represented by the column and the DEGs represented by the row. The top number in the cell represents the number of genes shared between the specific DMGs and DEGs, the bottom value represents the significance of such overlap determined using hypergeometric distribution against a whole genome background. N.S. stands for not significant (*p* > 0.05). A significant overlap, if occurring between up-regulated DEGs and DMGs with increased permissive marks (H3K4me3, H3K36me3, H3K27ac), or decreased repressive mark (H3K27me3 or H3K9me2), is considered as canonical gene activation and the cell is colored with green. A significant overlap, if occurring between down-regulated DEGs and DMGs with decreased permissive marks (H3K4me3, H3K36me3, H3K27ac), or increased repressive mark (H3K27me3 or H3K9me2), is considered as canonical gene repression and the cell is colored with light pink. Otherwise, a significant overlap is colored with yellow and represents a non-canonical gene regulatory event.

**Fig. 4:**
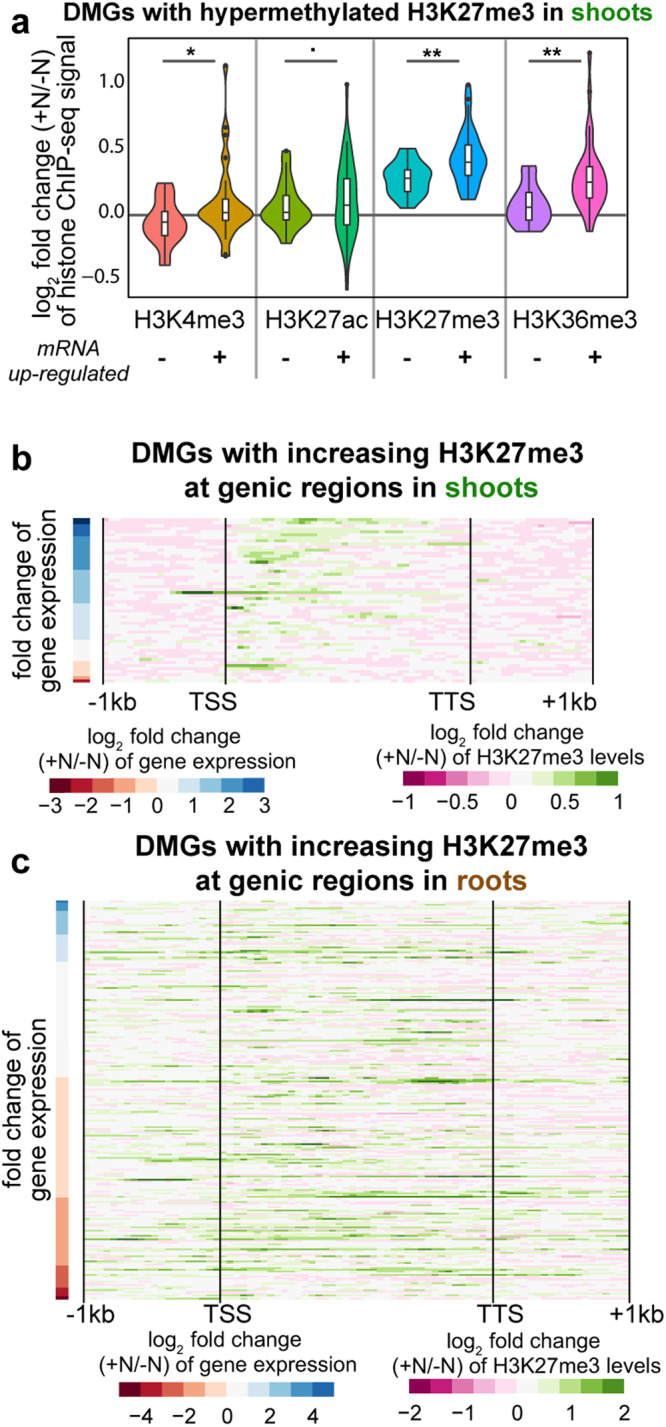
Up-regulated genes associated with increased H3K27me3 also show increased H3K4/K36 methylation, with H3K27me3 changes spreading over the transcribed region. **a** The log2 fold change of histone ChIP-seq coverage between +N conditions and −N conditions are plotted for the 57 genes with increased H3K27me3 in the shoots, specifically comparing between the 34 genes with increased mRNA levels and the remaining 23 genes that are not up-regulated. The log2 fold changes are plotted as violin plot using the R package ggplot2, and the statistical significance of differences between the two gene sets were determined using Student’s t-test. The *p*-value of the significant difference is indicated by asterisks: (•*p* < 0.1; *p < 0.05; **p < 0.01; ***p < 0.001; ****p < 0.0001). Overall, the up-regulated genes showed higher increases of H3K27me3, as well as increased H3K4me3 and H3K36me3. **b**, **c** Heatmaps showing the positional profile of changes of H3K27me3 from 1 kb upstream of transcription start site (TSS) to 1 kb downstream of transcription termination site (TTS) for genes with increased H3K27me3 at genic region in shoots (**b**) or in roots (**c**). Log2 fold change of H3K27me3 ChIP-Seq sequencing depth between +N and −N samples were plotted as heatmap with green color representing increased H3K27me3 in +N samples compared to −N controls and magenta representing decreased H3K27me3 in +N samples compared to −N controls. Each row in the heatmap represents one gene, and the expression level change of the gene is indicated by the color in the column on the left of the heatmap. Blue color represents up-regulation of transcript levels in +N samples compared to −N controls, while red color represents down-regulation. The genes are ranked with the most up-regulated genes at the top. Overall, the increased H3K27me3 is observed only in transcribed region in the shoots, while it spreads into the 1 kb promoter region in the roots. In the shoots, the most up-regulated genes are more likely associated with increased H3K27me3 distributed across the gene body from TSS to TTS.

To investigate the global relationship between epigenomic and transcriptomic changes in response to N supply, we compared the genes marked by dynamic histone modifications (DMGs) with genes showing differential expression (DEGs) (Fig. 3). We observed an overall pattern suggesting largely canonical gene activation or repression events, i.e., a gene whose transcript level is induced also displays increased permissive histone marks or decreased repressive histone marks, or vice versa. Indeed, for both shoots and roots, the permissive marks H3K4me3, H3K27ac and H3K36me3 in genic regions (Fig. 3a) or putative promoters (Fig. 3b) change concordantly with the changes of transcript levels, indicated by the significant overlap between up-regulated DEGs with genes showing increased H3K4me3, H3K27ac, or H3K36me3 (Fig. 3). In agreement with this, at whole genome level higher expressed genes are associated with higher levels of H3K4me3, H3K27ac, and H3K36me3 (Supplementary Fig. 4). Unexpectedly, the H3K27me3 mark displays both canonical and non-canonical patterns, depending on the organ and positional context (presence in the promoter *vs* the gene body). In the roots, increased H3K27me3 at the putative promoter region is associated with decreased gene expression, which is consistent with the notion that H3K27me3 functions as a repressive mark (Fig. 3b). In accordance with this, at the whole genome level, higher H3K27me3 around the promoter to TSS is associated with lower gene expression (Supplementary Fig. 4). In the shoots, however, a majority (34/57) of DMGs with increased genic H3K27me3 surprisingly showed up-regulated transcript levels in response to N (Fig. 3a). These 34 genes are enriched with nitrogen metabolism related GO terms (Supplementary Data 4) such as cellular amide metabolic process (adjusted *p* < 7E−11) and cellular nitrogen compound biosynthetic process (adjusted *p* < 5E−6) and include multiple ribosomal protein coding genes and an *NLP* transcription factor gene (*Solyc01g112190.3*, Fig. 2e). In comparison to the H3K27me3 hypermethylated genes that do not exhibit significant mRNA up-regulation (23/57), these 34 genes with H3K27me3 hypermethylation and concurrent N-induced mRNA expression showed a greater fold-change in H3K27me3 increase (Fig. 4a). This set of genes also show significantly higher levels of H3K4me3 and H3K36me3 (Fig. 4a), possibly indicating that the combination of activation marks (H3K4me3 or H3K36me3) and the repressive mark (H3K27me3) specify the transcriptional states of these up-regulated genes, though it is also possible that this epigenetic pattern is caused by mixing distinct cell types constituting the organs. Moreover, the increased genic H3K27me3 signal at up-regulated genes is located at the gene body without spreading into the promoter (Fig. 4b), and the DMGs that are most strongly up-regulated at the transcript level are associated with a dynamic H3K27me3 spreading toward the 3’ end of coding region (Fig. 4b). Interestingly, this non-canonical association of increased H3K27me3 with activated gene expression is only observed in the shoots but not in the roots. In the roots, the DMGs with increasing H3K27me3 at genic regions are associated with both down-regulated and up-regulated genes, while the increase of H3K27me3 levels is observed over the entire gene body and extends into promoter and downstream regions with no discernable relationship to the direction or magnitude of transcript level changes (Fig. 4c).

To determine whether this unexpected association between repressive histone mark and activated genes observed in shoots is specific for H3K27me3 or involves other repressive marks, we performed a follow-up ChIP-Seq for H3K9me2, a mark associated with transcriptional repression that can dynamically regulate genes under stress conditions^50^. The same shoot tissue samples as the previous ChIP-Seq analyses were used. We focused on the shoot based on the observation of non-canonical association only in this organ, as well as availability of tissue. We found that H3K9me2 signal was very low in genic space and mostly associated with unexpressed genes (Supplementary Fig. 4), in agreement with its known role in gene silencing and heterochromatin formation^51^. While N-responsive H3K9me2 regions were found to be associated with hundreds of genes, there was no significant overlap with differentially expressed genes (Fig. 3), which is distinctly different from H3K27me3. Additionally, there was no overlap between genes that gained H3K27me3 and those that gained H3K9me2 in response to nitrate supply. These results indicate that the association of repressive histone modifications with highly expressed genes in the shoots likely reflects a specific role for H3K27me3.

### Machine learning uncovers organ-specific rules for how the histone code can be used to predict changes in gene expression level

The concept of the histone code proposes that distinct combinations of histone modifications act together to direct downstream events that affect the transcriptional activity of associated genes^26^. With the recent growth of epigenomic data and availability of machine learning algorithms to interpret observations from complex biological systems, it is now feasible to investigate the rules of histone code by testing whether histone modification data could be used to train machine learning models to predict gene expression levels^52–56^. Here, we tested if the five histone modifications, individually or in combination, could be used to predict whether a gene is up-regulated, down-regulated, or non-responsive to a supply of nitrate. Moreover, we were interested in distinguishing the relative contribution of each histone mark in such predictions, and whether the observed rules are organ-specific or conserved between shoots and roots.

To this end, we first focused on the four histone marks assayed for both shoots and roots and calculated the following set of 16 epigenetic feature measurements (four values for each of the four histone marks) for every gene in the genome: (i) the level of the histone modification (i.e., normalized ChIP-seq signal) present in a genic region under −N conditions; (ii) the same measurement for +N conditions; (iii) the histone modification’s fold change in the genic region in +N relative to −N conditions; and (iv) a binary indication of presence or absence of dynamic histone modification in the 5 kb upstream sequence (putative promoter). In parallel, we classified all expressed genes in an organ into one of three gene sets: up-regulated, down-regulated, or unchanged. To achieve a more balanced classification that is desired in machine learning, we relaxed the statistical cutoff for detecting DEGs, for machine learning purpose only, to FDR < 0.05 without fold change cutoff. This led to classification of 6797 up-regulated and 6548 down-regulated genes in shoots, and 5724 up-regulated and 5362 down-regulated genes in roots, within a background of all genes tested by DESeq2 (i.e., ~24k genes with a measurable level of expression). Next, the 16 epigenetic features were used to train a random forest model to learn whether a gene is up-regulated, down-regulated, or unchanged by a supply of N using XGBoost^57^. Specifically, 80% of genes were used for training and 20% were used for testing in a rotating block round robin fashion. Over sufficient iterations, each gene was tested ten times and the mean prediction score for the probability of a gene being in the up- or down-regulated gene classes was calculated. The precision-recall curve and maximum F1 scores were calculated from the testing sets to measure the performance of the machine-learning models. The precision-recall curve and maximum F1 scores from a model trained with random predictors (i.e., randomly permutated epigenomic features) were also generated as a baseline.

**Fig. 5:**
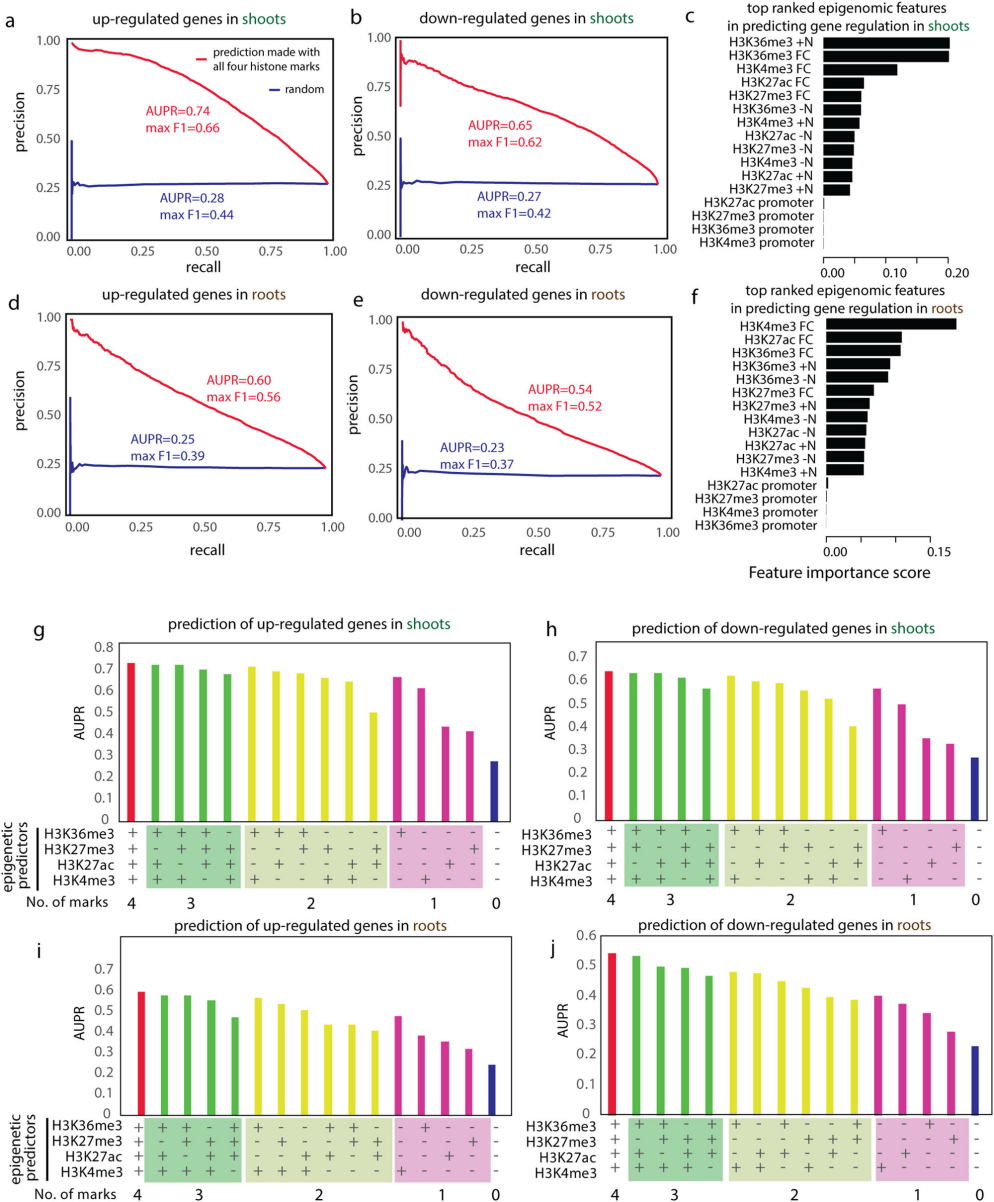
The combinatorial histone code was used to train machine learning models to predict gene regulation. Precision-recall curves were plotted for the prediction of up-regulated genes (**a**, **d**) or down-regulated genes (**b**, **e**) for shoots (**a**, **b**) or roots (**d**, **e**) using XGBoost machine learning models. Max F1 score [2 × (precision × recall)/(precision + recall)] and Area Under Precision-Recall curve (AUPR) were calculated as assessment of the performance of the machine learning models. The model trained with random predictors (i.e., randomly permutated epigenomic features) was also generated to provide a background for comparison (in blue curves). The feature importance scores for individual epigenetic features were plotted and ranked with the most important features at the top for shoots (**c**) and roots (**f**) separately. FC stands for fold change between +N samples and −N samples. **g**–**j** To determine the contribution of individual histone marks or combinations of histone marks in prediction gene regulation, the model training and testing was performed with predictors as: (i) all four histone marks; (ii) any three histone marks; (iii) any two histone marks; (iv) any one histone mark; or (v) random background with permutated epigenomic features, and the AUPR values were plotted and compared.

Our results showed that the combination of all four histone marks was able to predict the regulation of genes with a precision-recall curve that greatly outperformed the model trained with random predictors (Fig. 5a, b, d, e), with the best performance of AUPR = 0.74 and max F1 score = 0.66 for up-regulated genes in the shoots (Fig. 5a). In practical terms, of the top 1000 genes predicted by the machine learning approach to have the highest probability of being up-regulated in shoots, 955 were indeed up-regulated as supported by the RNA-seq data. This result could be explained by the histone code functioning as a causal factor to direct gene regulation, or by histone marks being modified co-transcriptionally as a result of gene regulatory events, or a combination of the two scenarios. The predictions in the shoots (Fig. 5a, b) performed better than in the roots (Fig. 5d, e), while the predictions for up-regulated genes (Fig. 5a, d) performed better than that for down-regulated genes (Fig. 5b, e).

Intriguingly, the four histone marks made unequal contributions to predicting gene regulation. Dynamic H3K27ac, canonically associated with gene activation, might be expected to perform as the best predictor for gene regulation in N response, based on the large number of DMGs (Fig. 1) and significant overlaps with DEGs (Fig. 3). However, the machine learning algorithm surprisingly arrived at a different conclusion: H3K27ac was not the best predictor of gene regulatory events at a genome-wide level (Fig. 5). In shoots, the genic H3K36me3 level and its fold change were the most important epigenetic features in predicting gene regulation (Fig. 5c), based on the feature importance score determined by XGBoost; in the roots, the fold-change of genic H3K4me3 was the most important component in predicting gene regulation (Fig. 5f). Indeed, in shoots, removing H3K36me3 from the epigenetic predictors had the biggest impact on the performance of prediction (Fig. 5g, h, green columns), while H3K36me3 as the sole epigenetic feature could predict gene regulation (Fig. 5g, h, pink columns) with a performance comparable to when the other three marks are used without it, for both up- (Fig. 5g) and down-regulated genes (Fig. 5h). In the roots, similarly, removing H3K4me3 from the epigenetic inputs had the largest effect in compromising the power of predictions (Fig. 5i, j, green columns), while H3K4me3 shows the best performance of the four when a single mark is used (Fig. 5i, j, pink columns). One possible explanation is that H3K4me3 and H3K36me3 are deposited co-transcriptionally with Pol II, thereby functioning as an informative proxy for predicting gene regulation. However, it is notable that one activation histone mark outperforms the others in predicting changes in gene expression depending on the organ, indicating that specific histone modifications (H3K36me3 in shoots and H3K4me3 in roots) may play vital roles in orchestrating gene regulatory mechanisms in an organ-specific manner.

Next, to determine the combinatorial effect of histone modifications, we tested all pairs of histone marks in their ability to predict gene regulation. As expected, the combination of H3K4me3 and H3K36me3 is the most powerful in predicting gene regulation (Fig. 5g–j, yellow columns). H3K27ac, although not the best performing predictor of gene regulation when used individually, performs reasonably well in combination with H3K4me3 or H3K36me3 (Fig. 5g–j). One possibility is that H3K27ac leads to a relaxed chromatin status which provides access to DNA for gene regulatory events to occur^58^, thus providing additional predicting power together with either H3K4me3 or H3K36me3.

**Fig. 6:**
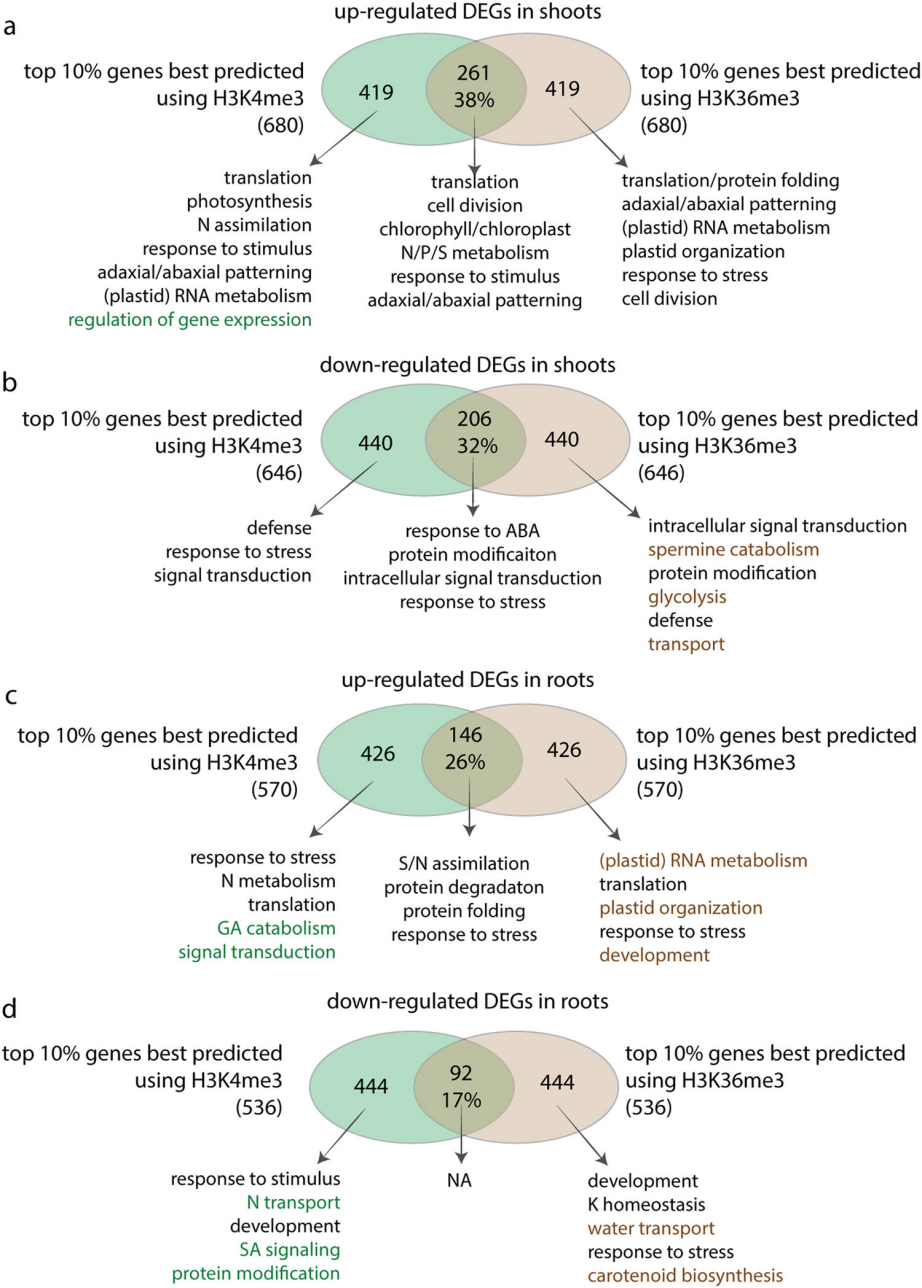
Partly overlapping but distinct sets of genes are best predicted by H3K4me3 or H3K36me3. The top 10% true positive genes that are correctly predicted by H3K4me3 or H3K36me3 as ranked by predicted probability score are represented by circles and venn diagrams are plotted to show the overlaps between gene sets, for shoots (**a**, **b**) and roots (**c**, **d**), and for up-regulated DEGs (**a**, **c**) and down-regulated DEGs (**b**, **d**), separately. The representative GO terms significantly enriched in the overlapped or distinct gene sets were determined with FDR adjusted *p* < 0.05 and trimmed using ReviGO to remove redundant GO terms. The GO terms in green highlights the specific biological processes uniquely identified for top 10% genes best predicted using H3K4me3 but not among the top 10% genes best predicted using H3K36me3, while GO terms in brown highlights the specific biological processes uniquely identified for top 10% genes best predicted using H3K36me3 but not among the top 10% genes best predicted using H3K4me3.

To investigate which subgroup of DEGs could be best predicted using specific epigenetic marks, we identified the top 10% true positive DEGs that are correctly predicted by individual epigenetic marks based on mean prediction score. We found that the DEGs that could be best predicted using H3K4me3 and those that could be best predicted using H3K36me3 are overlapping yet largely unique (Fig. 6). In the shoots, the top 10% of DEGs that could be best predicted by H3K4me3 or those by H3K36me3 share only 32–38% overlaps (Fig. 6a, b). In the roots, the overlapping sets are even lower, with 26% for up-regulated genes and 17% for down-regulated genes (Fig. 6c, d). These distinct gene groups share many similar enriched GO terms (Supplementary Data 5). For example, for up-regulated DEGs in the shoots, where the prediction performs the best (Fig. 5a), the ‘translation’ related GO terms are significantly enriched among the top 10% DEGs uniquely predicted by H3K4me3, as well as among the top genes uniquely predicted by H3K36me3 (Fig. 6a). On the other hand, some biological processes are only enriched among genes that are uniquely predicted using H3K4me3, but not among the top DEGs uniquely predicted by H3K36me3, and vice versa. For example, in the roots, up-regulated genes best predicted by H3K4me3 only are enriched with signal transduction, while up-regulated genes best predicted by H3K36me3 only are enriched with plastid RNA metabolism and plastid organization (Fig. 6d). Overall, we found that during a global reprogramming of gene expression, different histone marks function as the best indicator of gene regulation for distinct sets of genes, indicating gene specificity for different activation histone modifications.

We noticed that using our ChIP-Seq data for prediction of down-regulated genes did not perform as well as for up-regulated genes (Fig. 5) and wondered if the prediction was limited by incorporating information from only one repressive mark. To address this, the additional H3K9me2 ChIP-Seq data generated from shoot tissues were incorporated alongside data for the other four histone marks assayed in shoots in our machine learning approach to predict changes in gene expression in response to nitrate supply. The addition of H3K9me2 had a negligible effect when added to the other four marks for predicting up-regulated genes (Supplementary Fig. 5a; AUPR 0.736 vs 0.738) and only a slight improvement for down-regulated genes (Supplementary Fig. 5b; AUPR increases from 0.647 to 0.655). It also performed much worse than the other marks when used as the only information input for prediction (Supplementary Fig. 5, green lines). Given these observations alongside its low association with expressed genes and enrichment at silenced genes (Supplementary Fig. 4), it is possible that this mark is largely associated with non-expressed genes and thus has limited predicting power on dynamic gene expression.

## Discussion

### Distinct behaviors of different histone modifications during dynamic environmental responses

Our genome-wide analyses of histone modifications in tomato shoots and roots in response to N uncovered that each histone mark showed distinct dynamic behaviors in a variety of aspects: (i) the level of the dynamicity; (ii) the scope of gene targets; (iii) preference for transcribed vs regulatory regions; and (iv) association with gene activation or repression. H3K27ac is highly dynamic, affecting many gene loci in our study (Fig. 1a, b), which is in agreement with its fast turnover time^59^. H3K27ac changes are observed at both genic and intergenic regions in response to N supply (Supplementary Fig. 3), consistent with a role in facilitating transcription as well as marking enhancer regions as previously described^60,61^. The increase of H3K27ac is largely associated with gene activation (Fig. 3), which supports its canonical role in regulating actively transcribed genes^58^.

The repressive mark H3K27me3, which works in antagonistic manner with H3K27ac^62,63^, is also responsive at both genic and intergenic regions (Supplementary Fig. 3) and is more dynamic in roots than in shoots (Fig. 1c). While it is in general considered as a repressive mark associated with gene silencing^64^, which was confirmed by our analysis of this mark at the promoter regions (Fig. 3b, Supplementary Fig. 4), the *genic* H3K27me3 showed a surprising pattern of increasing at up-regulated gene loci in the shoots (Fig. 3a). This unconventional hypermethylation of H3K27me3, associated with gene activation events, showed many distinct features: (i) highly responsive H3K27me3 level in response to N evidenced by the greater fold-change (Fig. 4a); (ii) concurrent increases in activation marks H3K4me3 and H3K36me3 at the same loci (Fig. 4a); and (iii) spreading of H3K27me3 signal across the gene body from TSS to TTS while avoiding the promoters (Fig. 4b). This uncharacteristic enrichment of H3K27me3 at up-regulated gene loci was previously reported for nitrate transporter *NRT2.1*, possibly to restrict its high expression level^43^. In support of this, several *NRTs* (*Solyc05g006920.4, Solyc08g007060.4, Solyc08g077170.3*, and *Solyc05g006990.3*) are also marked with increased H3K27me3 in our dataset (Supplementary Data 1). Our genome-wide analysis thus expanded the previous report focused on a single gene locus^43^ to a genome-wide level and uncovered that in shoots six hours after a N-supply, increased H3K27me3 is largely observed at up-regulated gene loci. These genes include potential master regulator of N response (*NLP;* Fig. 2e), as well as genes involved in protein synthesis. Interestingly, the effect of H3K27me3 on gene expression in shoots appears to be highly positional, as higher H3K27me3 levels in the promoter region were associated with decreased gene expression (Supplementary Fig. 4). We speculate that the increased genic H3K27me3 may function as a mechanism to prevent over-induction of genes by N supply; it is also possible that H3K27me3 is deposited along the gene body of highly induced genes to repress ambiguous transcription initiation in the middle of the gene, or to place a limit on the rate of transcription occurring at the gene locus to minimize possible transcription errors. It is also possible that genic H3K27me3 affects transcript processing events such as RNA splicing, as proposed for another repressive epigenetic modification, DNA methylation^65^. A follow-up investigation of another repressive mark, H3K9me2, did not reveal a similar pattern, indicating that H3K27me3 may behave differently in some contexts than other marks associated with gene silencing. Indeed, it is known that the presence of bivalent H3K4me3 and H3K27me3 marks poises expression of development genes in animals^66^ and is associated with the transcriptional activation of stress-responsive genes in plants^67,68^.

Finally, H3K4me3 and H3K36me3 are observed to significantly change at a smaller set of gene loci (Fig. 1), which is consistent with their relative stable nature at this time scale^48^. Dynamic H3K4me3 and H3K36me3 marks, in contrast to H3K27me3/ac, are mostly limited to genic regions (Supplementary Fig. 3) and strictly associated with active gene transcription (Fig. 3), concordant with their known roles in marking genes actively transcribed by Pol II^69,70^ (Fig. 3, Supplementary Fig. 4). Interestingly, dynamic changes of different histone modifications are targeted to genes involved in vastly different biological processes (Fig. 1d, e). This invites an intriguing question of how the target specificity is achieved. It could be speculated that downstream of N signaling, various TF partners recruit different epigenetic regulators (for example, a bZIP transcription factor interacts with a HAT^71^) to direct them to the specific set of target gene loci relevant to a particular biological process (e.g., photosynthesis) to regulate their transcriptional states, thus impacting the activity of the specific biological processes.

Examining the epigenetic and transcriptional regulation of N-relevant TF and enzymes revealed complexity of how multiple histone marks work in concert to regulate gene activity^72,73^. When examining a specific and well-studied biological process, N assimilation (Fig. 2), we found that the binary classification of gene regulation events into up-regulated or down-regulated classes is further complicated by order-of-magnitude higher diversity of histone code. For example, an up-regulated N assimilation gene could be marked with increased H3K27ac (e.g., GS2 and *GSR2* in the shoots [Fig. 2d]), or increased H3K36me3 (*GLT1* [Fig. 2d]). Across the genes analyzed (Fig. 2), ~40% are regulated by more than one dynamic histone modification. The multiple dynamic chromatin modifications observed at a given gene locus could result from recruitment of distinct epigenetic regulators by the same or different transcriptional regulation pathways, all of which act downstream of N signaling. Those genes that are a target of multiple histone modifications, such as *NIA* and *NIR*, may reflect hotspots of regulation from multiple transcriptional regulatory inputs, though it is also possible that this observation could result from heterogeneity of cell types, with the changes of different histone marks occurring in different subpopulations of cells in the organ sampled.

In our study, we focused on analyzing the histone marks at the promoter and genic regions. For the histone marks in downstream and intergenic regions, the signals appear to be noisier. Moreover, the dynamic islands at the 5 kb downstream regions displayed little relevance to changes in gene expression. The dynamic islands at the intergenic regions on the other hand, could be highly interesting as it might be relevant to enhancers or long-range interactions, however, it is in generally challenging to assign intergenic regions to genes that they should influence. Further investigation is needed to dissect the effects of intergenic histone marks on the transcriptional regulation of genes.

### Machine learning as a tool to interpret the complex relationship between epigenetic states and transcriptional regulation

Recently, with the increasing amount of publicly available epigenomic data and advances in applying machine learning algorithms to decipher complex biological systems, histone modifications have been used to train machine learning models to predict gene expression levels in different cell types in mammalian systems^52–56^. Our study is unique in that: (i) it focuses on predicting dynamic changes in response to environmental stimulus rather than steady state gene expression; (ii) it uses in planta data; and (iii) machine learning models were built for shoots and roots separately, using epigenomic and transcriptome data generated from the same organs, thus providing interesting yet otherwise hidden insights such as the organ specificity of the machine learning predictions. The machine learning approach showed great potential to provide a new perspective by helping to interpret the complex relationship between epigenetic state and dynamic gene regulation. While H3K27ac has a dramatic dynamic change in response to N at specific gene loci (Fig. 1) and is well associated with gene regulation (Fig. 3), it performs poorly as a predictor of gene activation or repression at the level of the whole genome. It could be that H3K27ac primes genes to enable their activation as previously described for light response^74^, therefore being a required but not sufficient mark for gene activation, which might have limited its predicting power. H3K4me3 and H3K36me3, while showing less dramatic changes compared to H3K27ac at specific gene loci, are more accurate at a genome-wide level in predicting whether a gene is up-regulated or down-regulated. The limitation of our study lies in the fact that the machine learning models, while rather powerful in discerning hidden patterns that are not easily detected by correlation or regression, do not necessarily inform causal relationship between histone modifications and gene transcriptional regulation. Therefore, our results could be explained in both directions of cause-effect relationships: (i) this could reflect that H3K4me3 and H3K36me3 directly affect gene regulation; indeed, it has been observed that the loss of H3K36me3 and the related histone methyltransferase led to altered transcriptional response to N in Arabidopsis^46^; or (ii) H3K4me3 and H3K36me3 are deposited co-transcriptionally with Pol II in roots and in shoots, respectively, thereby functioning as an effective proxy for gene regulation^69,70^.

In our study, the prediction of up-regulated genes was more successful than prediction of down-regulated genes. Incorporating data from an additional repressive mark, H3K9me2, did not notably improve the prediction of down-regulated genes (Supplementary Fig. 5). It is possible that the regulation events are better explained by another mark, such as DNA methylation, which we did not assay; many important genes involved in DNA methylation are differentially expressed in response to N supply (Supplementary Data 6), including *CMT3* which has previously been connected to regulation of *NIA2* in Arabidopsis^75^. It is also possible that down-regulation is more dependent on events occurring outside of nuclear transcriptional activity, such as regulation of mRNA degradation.

### Organ specificity of dynamic histone modifications

It is well known that histone modifications are associated with cell identity and differentiation during and after organ development^76–78^. Our study now adds new dimensions in the organ specificity of epigenetic modifications by revealing that dynamic changes in histone modifications in response to an external signal are also organ-specific (Fig. 1c). The difference observed between roots and shoots are most likely due to functional difference between the two organs, rather than merely a time lag of signaling between the two organs. Indeed, it was shown previously that within two hours of nitrogen treatment, the shoots and roots have almost equal numbers of genes differentially regulated^16^. In fact, genes involved in nitrate response and nitrate transport are induced within 5 min of roots experiencing a difference in N levels^16^. Therefore, at our timing of sampling (6 h after treatments), it is unlikely that the transcriptional difference between shoots and roots is purely due to time lag, as opposed to being governed by the different roles of shoots and roots in N uptake, assimilation, and signaling. Moreover, our epigenomic data showed that shoots and roots have similar numbers of genes displaying dynamic histone acetylation in response to N treatment at the time point we sampled, and that shoots and roots share N assimilation genes that are dynamically modified, but N transporter genes are specifically modified in the roots, which also supports an organ functional difference with roots being the main organ for nutrient uptake.

In addition, our study found that different marks have different levels of organ specificity in response to N supply, with H3K27ac being the least organ-specific, and H3K27me3 being the most organ-specific (Fig. 1c). It is possible that histone acetylation is more likely to occur at housekeeping genes whose regulation is less organ-specific, while H3K27me3 occurs at genes that are expressed and regulated in an organ-specific manner^74^. Overall, it is reasonable to speculate that perceived and transduced signals (in this case, perception of N supply by roots, which is known to induce systemic signals^79^) cause different epigenetic machinery in the two organs to direct changes at the chromatin level. Alternatively, the signaling cascade and the responsive epigenetic machinery could be similar between shoots and roots, with existing baseline differences in the chromatin landscape between shoots and roots contributing to the organ-specific chromatin changes we observed. A combination of the above two models—a different universe of epigenetic regulators active in each organ working on the organ-specific basal chromatin landscape at target loci—could also be the case. Indeed, in our transcriptome dataset, we observed a group of epigenetic regulator genes that are regulated by N supply in either shoots, or roots, or in both organs (Supplementary Data 6).

Our most surprising observation, however, was that the specific crosstalk between histone modifications and gene regulation, i.e., the rules of how histone modification influences gene regulation, or vice versa, are also heavily dependent on the organ context. We uncovered an association between increased genic H3K27me3 with up-regulated genes as a unique phenomenon observed in the shoots but not in roots (Figs. 3, 4). Moreover, using a machine learning approach to discern the genome-wide relationship between dynamic histone modifications and gene regulation, we found distinct rules governing this relationship in the two organs; in the shoots, H3K36me3 is the most informative in predicting gene regulation and is dominant over other marks in power of prediction to the degree that using only this mark performs similarly to using the other three combined (Fig. 5c, g, h). In the roots, however, H3K4me3 is the most informative in predicting gene regulation events (Fig. 5f, i, j). It is possible that in the shoots, histone methyltransferases responsible for H3K36me3 (like SDG8) are recruited alongside transcription initiation and elongation machinery while in the roots the predominant recruited histone methyltransferases catalyze H3K4me3 deposition in transcribed regions. Whether this is a feature unique to response to N or a more universal mode-of-action underlying organ-specific transcriptional reprogramming in response to environmental stimuli requires further study. Further, whether these rules translate to other flowering plants outside of tomato remains to be determined. Moreover, our approach for prediction of gene regulation performed better in shoots than that in the roots. This may indicate that additional histone marks beyond those we measured make important, non-redundant contributions to root response to N supply and would be needed to predict gene expression more accurately. It could also reflect differing degrees of post-transcriptional regulation affecting mRNA stability and turnover in the two organs, which might impair the ability of epigenetic marks to predict transcript levels. Overall, when one talks about the general rules of how a specific histone modification affects gene transcription, it is important to note that the specific mode of regulation—when and where it is added or removed as well as how it is perceived or acted upon by downstream regulators—are likely dependent on organ- or tissue-specific contexts.

## Conclusions

Our analysis of epigenomic data from shoots and roots uncovered organ-specific chromatin dynamics associated with transcriptional reprogramming, indicating that downstream of nitrate signaling distinct epigenetic machinery functions in each organ to modify histones at functionally relevant gene loci and manifest proper transcriptional response. We found a non-canonical role of H3K27me3 in modifying a group of actively transcribed genes, possibly to prevent over-expression. Using machine learning approaches, we found that while gene regulation could be best predicted using the combination of all four histone marks assayed, H3K36me3 and H3K4me3 are the most informative in predicting gene regulation, in shoots and roots respectively. In summary, our integrated epigenomic and transcriptomic study provides evidence to support the organ specificity of chromatin modifications during plant environmental response: most dynamic histone modifications we observed are organ-specific in nature, and the relationship between the histone code and transcript level changes are also governed by organ-specific rules.

## Methods

### Plant growth and nitrogen treatment

Tomato (*Solanum lycopersicum*; cultivar M82) seeds were first sterilized with 20% bleach and then sown on plates (1% agar; ½ MS) to germinate in a growth chamber with 16 h day (100 µmol/s/m^2^; 24 °C) and 8 h night (0 µmol/s/m^2^; 20 °C) cycle for eight days (Supplementary Fig. 1). Germinated seedlings were then transferred to a hydroponic system to allow convenient treatment with nitrogen and sampling of shoot and root tissues (Supplementary Fig. 1). Specifically, the seedlings were grown in 1 L plastic container with hydroponic growth medium^80^ (1.2 mM KNO_3_, 0.8 mM Ca(NO_3_)_2_, 0.2 mM KH_2_PO_4_, 0.2 mM MgSO_4_, 50 µM KCl, 12.5 µM H_3_BO_3_, 1 µM MnSO_4_, 1 µM ZnSO_4_, 0.5 µM CuSO_4_, 0.1 µM H_2_MoO_4_, 0.1 µM NiSO_4_, and 10 µM Fe-EDDHA, pH 6.0) under common greenhouse conditions. The growth medium was renewed every 2–3 days. After 14 days, the plants were treated with N-free starvation medium, wherein the KNO_3_ and Ca(NO_3_)_2_ in growth medium were replaced with equimolar KCl and CaCl_2_, respectively (Supplementary Fig. 1). After 4 days in the starvation medium, the plants were either treated with fresh starvation medium (−N treatment) or treated with fresh growth medium (+N treatment) (Supplementary Fig. 1). Six hours after the +N/−N treatments, root and shoot tissues were harvested and flash frozen in liquid nitrogen for total RNA extraction, as well as fixed in 1% formaldehyde and flash frozen in liquid nitrogen for ChIP analysis, in three biological replicates with tissues from four plants pooled as one biological replicate. Additionally, plants were grown in the +N/−N treatment medium for 6 days for physiological phenotyping (Supplementary Fig. 1).

### Chlorophyll analysis

Leaves from individual seedlings were frozen in liquid nitrogen and ground to fine powder using MiniG (SPEX, Metuchen NJ). 15 to 45 mg of powdered tissue was weighed and then suspended in 1 ml methanol, and the suspension was rotated for 10 min at room temperature to extract chlorophyll. The suspension was centrifuged for 5 min at 14,000 rpm, and 800 µl supernatant was transferred to a 1.5 ml tube for measurement. Absorbance at 750 nm, 665 nm, and 652 nm was measured using Nanodrop One (Thermo Fisher Scientific, Wilmington DE). The chlorophyll content was calculated following the protocol of Porra et al.^81^.

### Chromatin immunoprecipitation sequencing (ChIP-Seq)

The chromatin immunoprecipitation (ChIP) was performed according to previously published protocols^82,83^ with modifications for tomato roots and shoots. Briefly, two grams of tissue were fixed with 1% formaldehyde by applying vacuum at 700 mmHg for 25 min. Fixation was terminated by adding 2 M glycine to a final concentration of 0.125 M and vacuum application for 5 min. Formaldehyde-fixed tissues were ground in liquid nitrogen and nuclei were isolated following the protocol of Gendrel et al.^82^. Isolated nuclei were sonicated using a Bioruptor Pico on high power setting (Diagenode, Denville NJ) for at least ten sonication cycles (each cycle includes 30 s of sonication followed by a minute of break) to prepare chromatin samples. An aliquot of the chromatin samples was kept as input DNA to provide a background of chromatin samples without immunoprecipitation; it has been reported that using input DNA and using H3 as controls are comparable^84^. The rest of chromatin samples were immunoprecipitated using Protein A dynabeads (Life Technologies, Carlsbad CA) coated with antibodies against H3K4me3 (Millipore Sigma 07473), H3K27ac (Millipore Sigma 07360), H3K27me3 (Millipore Sigma 07449), or H3K36me3 (Abcam ab9050), to pull down genomic DNA associated with specific histone modifications. Immunoprecipitation with no antibody was included as a negative control to measure the level of non-specific pull down. The precipitated chromatin fragments were reverse-crosslinked, and the associated DNA was purified. As internal quality control, we performed ChIP-qPCR to measure the fold enrichment of ChIP DNA over the no antibody control at select gene loci; all ChIP DNA were enriched >50-fold over the no antibody control in our study, indicating satisfactory ChIP quality. Using NEBNext dual index library kit (New England Biolabs, Ipswich MA), next-gen sequencing libraries were generated for ChIP DNA samples and corresponding input DNA samples as background controls. A total of 60 libraries, consisted of two treatments (+N and −N), two organs (shoots and roots), for four histone marks plus input DNA, and in three biological replicates, were sent to Novogene (CA, USA) for paired-end 150 bp sequencing in NovaSeq platform (Illumina, San Diego CA) to generate an average yield of 40 million read pairs per library. H3K9me2 (ab1220) ChIP-Seq was performed similarly to above, with 12 total libraries for two treatments (+N and −N) in shoots for input chromatin and H3K9me2 pulldown.

### ChIP-seq data analysis

Raw ChIP sequencing reads were trimmed using Cutadapt^85^ and aligned to *Solanum lycopersicum* genome build 4.0 (International Tomato Genome Sequencing Project) using Bowtie2^86^. The resulting BAM files were sorted by read name using SAMtools^87^ and converted to BED format using the bamtobed command of BEDTools^88^. SICER^89^ was used to detect genomic regions with significant enrichment of histone mark compared to the input DNA control. SICER-df was used to identify genomic regions (islands) that are differentially marked between nitrate-treated samples and controls, with the following options: window size = 200 bp, gap size = 200 bp (except for H3K27me3: gap size = 600 bp), effective genome fraction = 0.9 and false discovery rate (FDR) threshold = 0.05. Significant dynamic islands in response to N were then filtered for fold-change of at least 1.5 between the +N and −N conditions and used for further analysis. We used this cutoff, similar to other studies^39,46,64,90^, because: (1) compared to RNA-Seq analysis that samples multiple copies of a transcript in a cell, histone ChIP-Seq samples only one copy of genomic DNA from a cell, therefore a smaller dynamic range is expected; (2) the nitrate treatment is a transient treatment of only six hours, therefore, we expect to capture minor but dynamic changes in epigenome; and (3) histone methylation compared to histone acetylation is relatively stable during the time frame of our treatment (in hours^48^). Next, the “closest” tool in the BEDTools was used to locate dynamic islands to gene bodies, upstream promoter (5 kb), or downstream (5 kb) of annotated genes or classify them as intergenic (none of the above). Differentially modified genes (DMGs) were defined as genes with a significantly dynamic island for at least two of three biological replicates. The “genomecov” tool in BEDTools was used to calculate the genome coverage from ChIP-seq data, which was then processed with a custom python script to generate positional ChIP-Seq depth along genes. Specifically, each gene was partitioned into 80 bins, including 20 bins representing the 1 kb promoter sequence upstream of the transcription start site (TSS), 40 bins representing the transcribed region of gene from the TSS to transcription termination site (TTS), and 20 bins representing 1 kb sequence downstream of TTS. ChIP-seq depth for each bin was summarized by calculating the average and then normalized as the number of fragments per million for visualization. Reproducibility in the ChIP-Seq replicates (Supplementary Figs. 6, 7) was measured using the cor() function in R to determine the genome-wide Pearson correlation for normalized genic histone mark signal which was plotted using corrplot^91^.

Arabidopsis homologs for *S. lycopersicum* proteins were determined by BLAST against Araport11 annotations with an E-value cutoff of 1e-07 for significance. ShinyGO^92^ was used to identify significantly enriched gene ontology (GO) terms in each gene set with an FDR cutoff of 0.05.

### RNA-Seq

Tomato root and shoot tissues were ground in liquid nitrogen and total RNA was extracted using mirVana kit (Invitrogen, Carlsbad, CA) following the manufacturer’s protocol for total RNA extraction. Turbo DNase (Invitrogen, Carlsbad, CA) was used to remove contaminating DNA and then the quality of extracted total RNA was analyzed using Bioanalyzer (Agilent, Santa Clara, CA) at the Purdue Genomics Core. The RNA integrity numbers (RINs) of total RNA samples were generally >9.1 for root samples and >7.3 for shoot samples, indicating high quality of total RNA. In total, 12 RNA samples (three biological replicates for two treatments in two organs) were sent to Novogene (CA, USA) for sequencing in paired-end 150 bp format using NovaSeq platform (Illumina, San Diego CA) to generate ~20-25 million read pairs per library. Cutadapt^85^ was used to trim adaptors and low-quality bases, and to discard short reads. Trimmed reads were then mapped to the *S. lycopersicum* genome build 4.0 using Tophat2^93,94^. The gene counts were generated from mapped reads using htseq-count^95^. Finally, differentially expressed genes (DEGs) were determined using DESeq2^96^ to compare +N samples with −N controls in either shoots or roots with statistical cutoffs of FDR < 0.015 and fold-change >2. Functional enrichment of DEGs were determined by gene ontology enrichment analysis using ShinyGO^92^.

### Machine learning

All expressed genes (~24k genes in shoots and roots, separately) were assigned to up-regulated, down-regulated, or unchanged gene groups based on a cutoff of FDR < 0.05 determined by DESeq2. This led to 6797 up-regulated and 6458 down-regulated genes in shoots, and 5724 up-regulated and 5362 down-regulated genes in roots. For each gene, the following epigenomic features were included as predictors: (i) twelve quantitative predictors: levels of each histone mark under −N or +N conditions were calculated as the mean of three biological replicates of average ChIP-seq coverage depth through the gene body along with the fold-change between the two conditions; (ii) four qualitative predictors: dynamic histone modifications at the putative promoter regions (5 kb upstream of TSS) were indicated by binary values (presence *vs* absence). These 16 values were used to predict gene regulation (up-regulated, down-regulated, or unchanged) using XGBoost^57^ with the “gbtree” booster and “multi:softprob” objective for multiclass prediction. 80% of genes were used for training while 20% were used for testing in a rotating block fashion across multiple gene matrices with randomly permutated gene order. Each gene is present in the testing group 10 times, and the mean prediction value for probability that the gene was up-regulated or down-regulated was determined. To measure the performance of the prediction, an in-house python script was used to determine precision, recall, and F_1_ score (the harmonic mean of precision and recall) at a stepwise descending cutoff of prediction value. The observed true positive (TP), false positive (FP), and false negative (FN) genes at each cutoff were used to calculate the performance metrics as follows: precision = TP/(TP + FP), recall = TP/(TP + FN), and F_1_ = 2⋅(precision·recall)/(precision + recall). The area under the precision recall curve (AUPR) was determined using the “AUC” function of DescTools (https://cran.r-project.org/web/packages/DescTools/index.html). The above approach was also used across various input datasets with omission of data for one or more histone modifications. The top 10% of DEGs predicted by a mark were determined from predictions made using only H3K4me3 or H3K36me3 data, ranking all true positive up-regulated or down-regulated genes by the determined probability from the machine learning they belonged to that regulated gene classification. The exclusive and overlapped gene sets for DEGs associated with the two marks in each organ were used for GO term enrichment using a GO term background populated from the closest *A. thaliana* homolog for each tomato gene using an in-house python script^97^ adapted for *S. lycopersicum* with an FDR < 0.05 cutoff for term enrichment.

### Statistics and reproducibility

All RNA-Seq and ChIP-Seq experiments were performed using three independent biological samples. Specific FDR and fold-change cutoffs are described in the relevant subsections. For significance testing of gene set overlaps, *p* was determined using a hypergeometric test in R.

### Reporting summary

Further information on research design is available in the Nature Portfolio Reporting Summary linked to this article.

### Supplementary information

Supplementary Figures

Description of Additional Supplementary Data

Supplementary Data 1

Supplementary Data 2

Supplementary Data 3

Supplementary Data 4

Supplementary Data 5

Supplementary Data 6

Supplementary Data 7

Supplementary Data 8

Supplementary Data 9

Reporting Summary

## Data Availability

All raw and processed sequencing data generated in this study have been submitted to the NCBI Gene Expression Omnibus (GEO; https://www.ncbi.nlm.nih.gov/geo/) under accession number GSE196887.
