# Supplementary material for: Organ-specific characteristics govern the relationship between histone code dynamics and transcriptional reprogramming during nitrogen response in tomato
Source: Commun Biol. 2023 Dec 4;6:1225. doi: 10.1038/s42003-023-05601-8 (PMC10694154; doi:10.1038/s42003-023-05601-8)
Supplement: Supplementary file 1 — Supplementary Figures [file 42003_2023_5601_MOESM1_ESM.pdf]

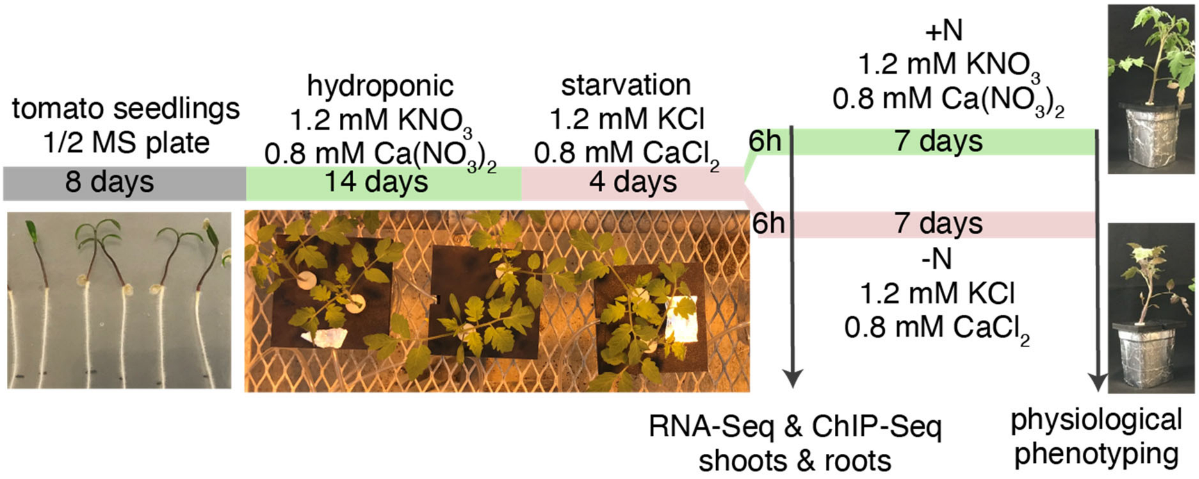

Supplementary Figure 1. Experimental scheme illustrating the treatment and profiling of organ-specific responses to nitrate. Tomato plants (*Solanum lycopersicum*, cultivar M82) were germinated on plates and then grown in hydroponic conditions. Three-week-old tomato seedlings were then treated with 2.8 mM NO_3^- (+N), or with 2.8 mM Cl^- (-N) as control, after an N-starvation period. The physiological phenotyping of biomass, shoot-to-root ratio, and chlorophyll content were performed after seven days of treatment. Shoot and root tissues were harvested six hours after the treatment for RNA-Seq and ChIP-seq analyses.

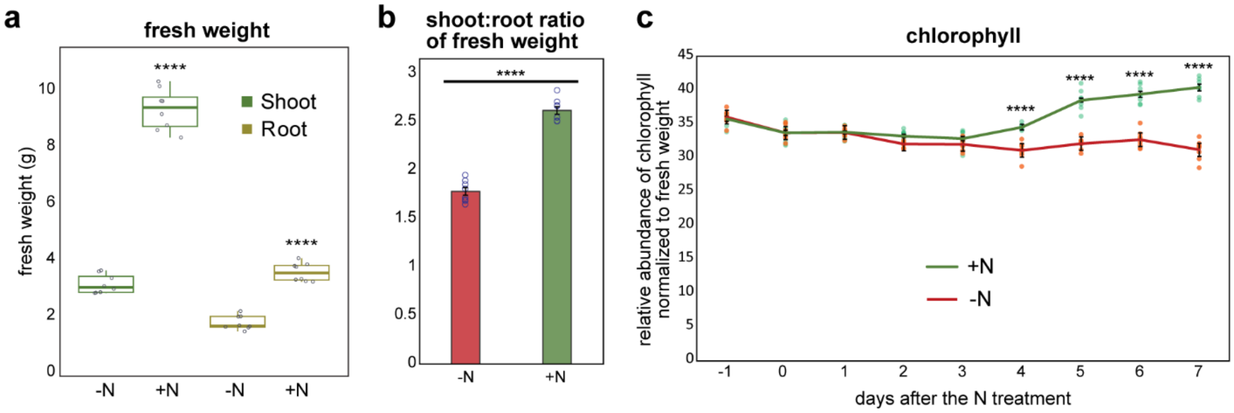

Supplementary Figure 2. The nitrogen treatment led to increased biomass (a), greater shoot-to-root ratio (b), and higher chlorophyll content (c). Error bars represent standard error of mean (N=8). Two tailed Student's t-test was performed to compare -N and +N samples, with the significance represented by asterisks: (•:p<0.1;*:p<0.05;**:p<0.01;***p<0.001; ****:p<0.0001).

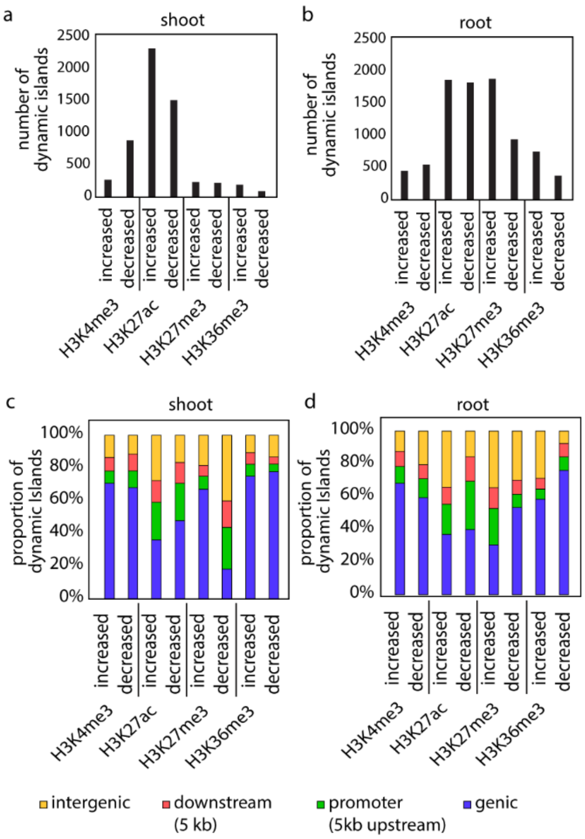

Supplementary Figure 3. The nitrogen treatment leads to dynamic changes of histone modifications at hundreds to thousands of genomic loci (dynamic islands) that are distributed at genic regions, putative promoters (5kb upstream), or 5kb downstream of annotated genes, as well as intergenic regions. (a-b) Number of dynamic islands, which are genomic regions associated with H3K4me3, H3K27ac, H3K27me3, or H3K36me3 modification that are significantly different between +N and -N conditions (FDR<0.05 and fold-change>1.5, average number of dynamic islands across 3 replicates), are plotted for the four histone marks in roots (a) or in shoots (b). "Increased" indicates that the histone modification is higher in +N conditions compared to the -N controls, and *vice versa*. (c-d) Percentage of dynamic islands that colocalize with different genome features are plotted, including genic (transcribed) regions, putative promoter regions (5kb upstream), regions 5kb downstream of annotated genes, as well as intergenic regions.

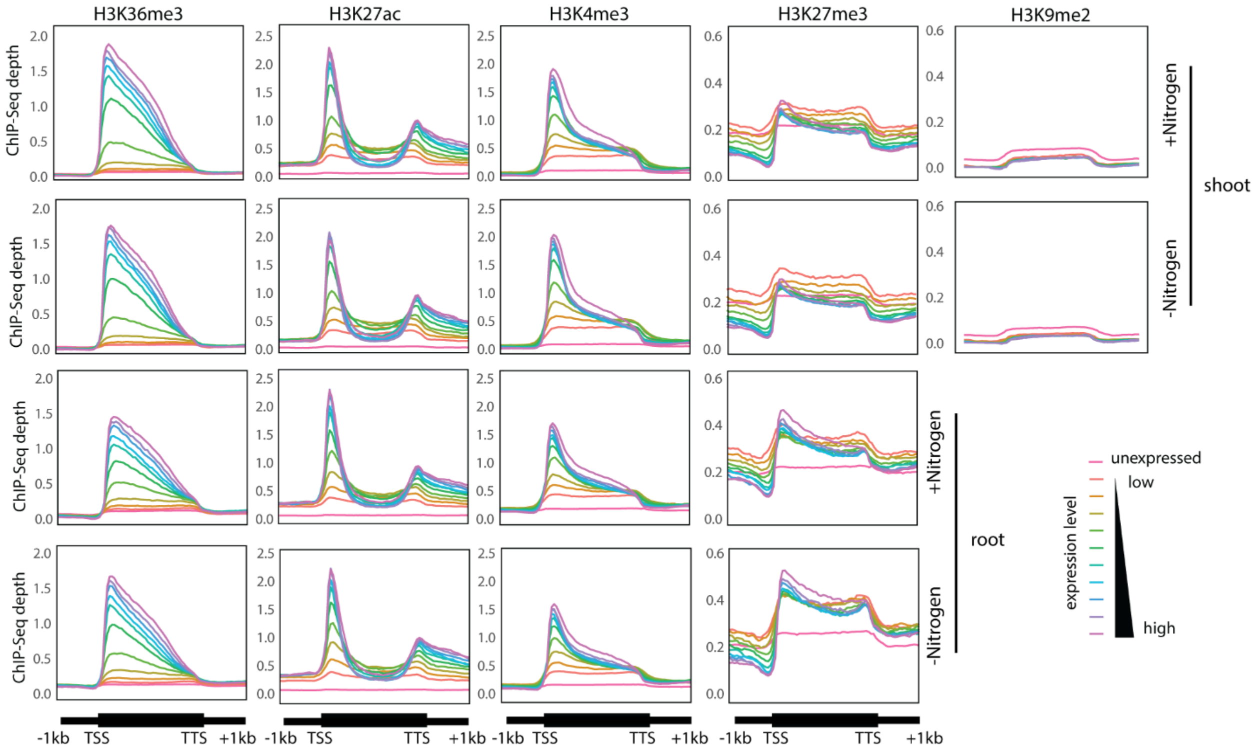

Supplementary Figure 4. Relationship between ChIP-seq signal in genic regions for histone modifications in shoot and root and expression level. Mean normalized FPM (fragments per million) histone mark coverage from ChIP-seq across three replicates at annotated genes was determined within the promoter region (upstream 1kb of the TSS), the downstream region (1kb from the TTS), and across the gene body normalized by gene length. Transcript length normalized gene expression in each organ and N supply condition was used to divide genes into expressed genes in ten equally sized groups ranked by expression along with a set of unexpressed genes (no counts observed). The median coverage across all genes in an expression tier was graphed. In general, a clear association with expression level of the gene was observed for H3K4me3, H3K27ac, and H3K36me3 levels in the gene body region. For H3K27me3, higher levels in the promoter were associated with low gene expression, while higher levels of H3K27me3 in the gene body region downstream of the TSS were associated with both low expression genes and very high expression genes. However, H3K27me3 levels generally decreased for high expression genes

across the gene body toward the TTS and were lower in the downstream region. Signal for H3K9me2 in genic space was mostly found to be associated with unexpressed genes.

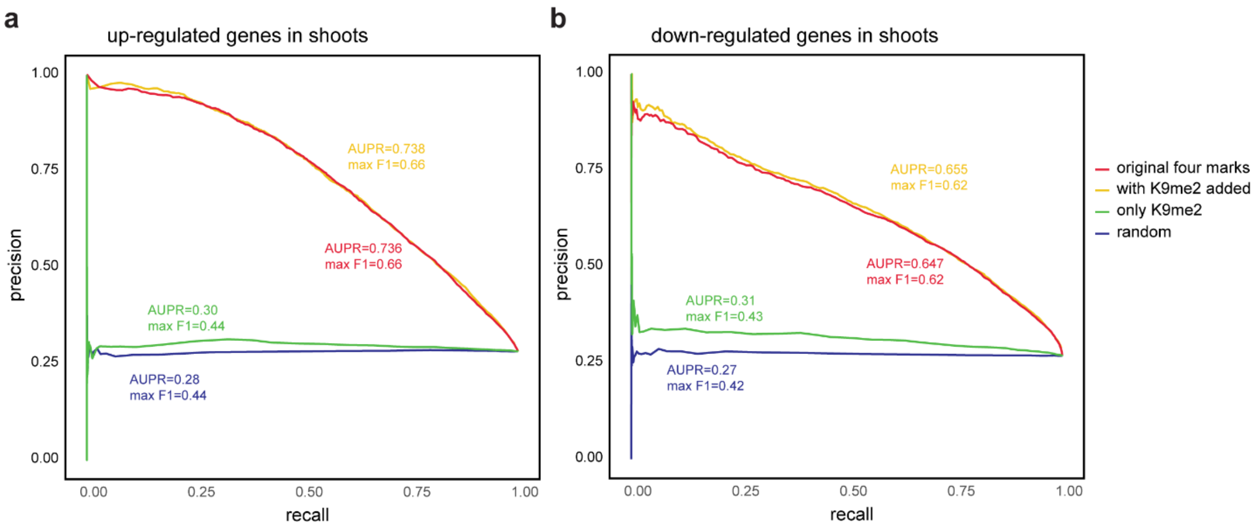

Supplementary Figure 5. Incorporating H3K9me2 data into machine learning predictions results in minor to negligible improvement. Precision-recall curves were plotted for the prediction of up-regulated genes (a) or down-regulated genes (b) in shoots. Results from the incorporation of H3K9me2 data to supplement the other four other marks (yellow) are graphed alongside the results previously obtained from the four marks as shown in Fig. 5 (H3K4me3, H3K27ac, H3K27me3, H3K36me3; in red) or random predictors (blue) as well as results obtained from using H3K9me2 alone (green) along with AUPR and max F1 scores (see Fig. 5 for more detail).

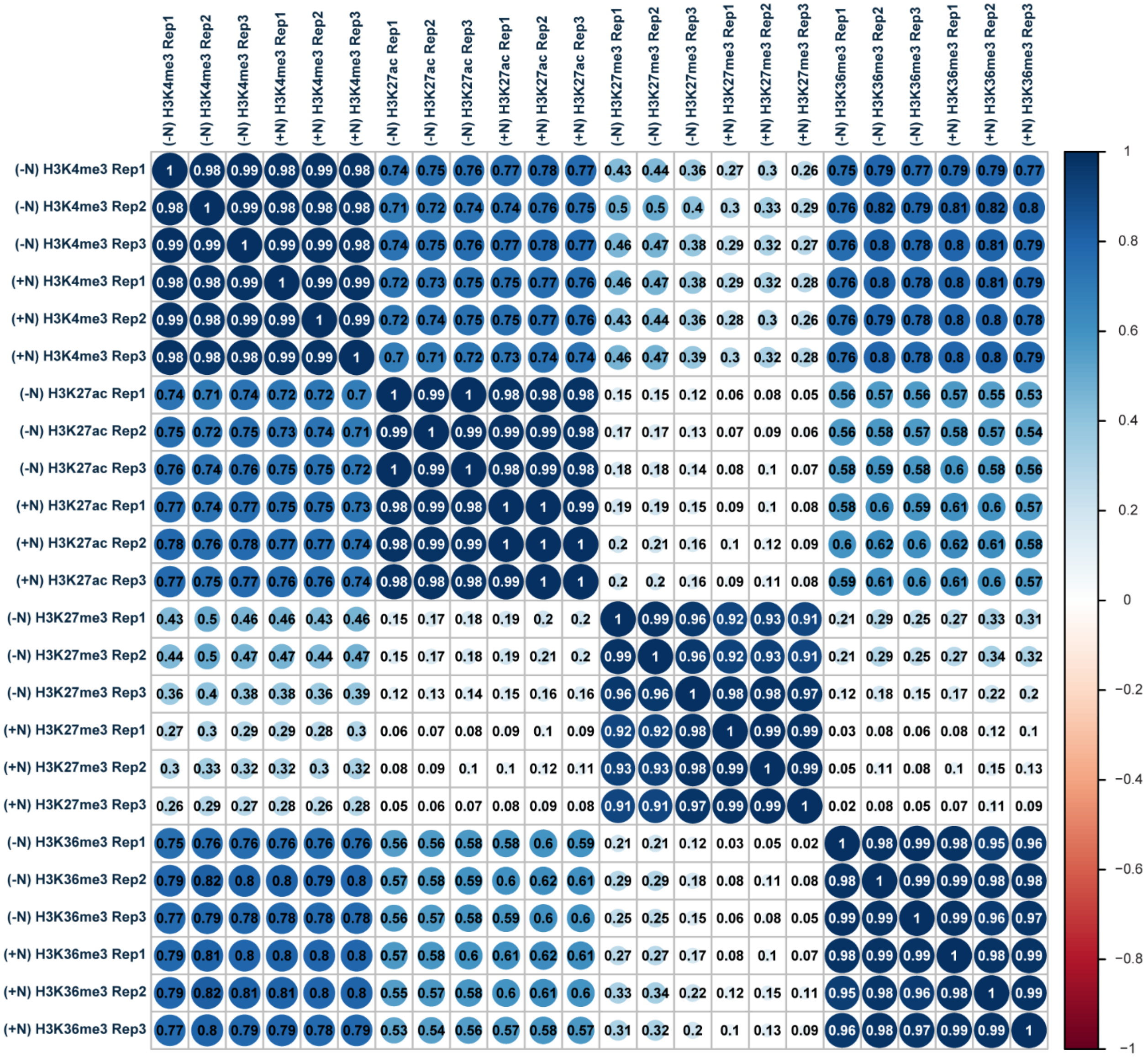

Supplementary Figure 6. Correlation between genic ChIP-Seq signal between replicates in root. Pearson correlation for ChIP-Seq signal (normalized by fragments per million and gene length, as used for input in machine learning predictions) for all annotated coding genes across all ChIP-Seq replicates in tomato root. Note that values ≥0.995 appear as 1.

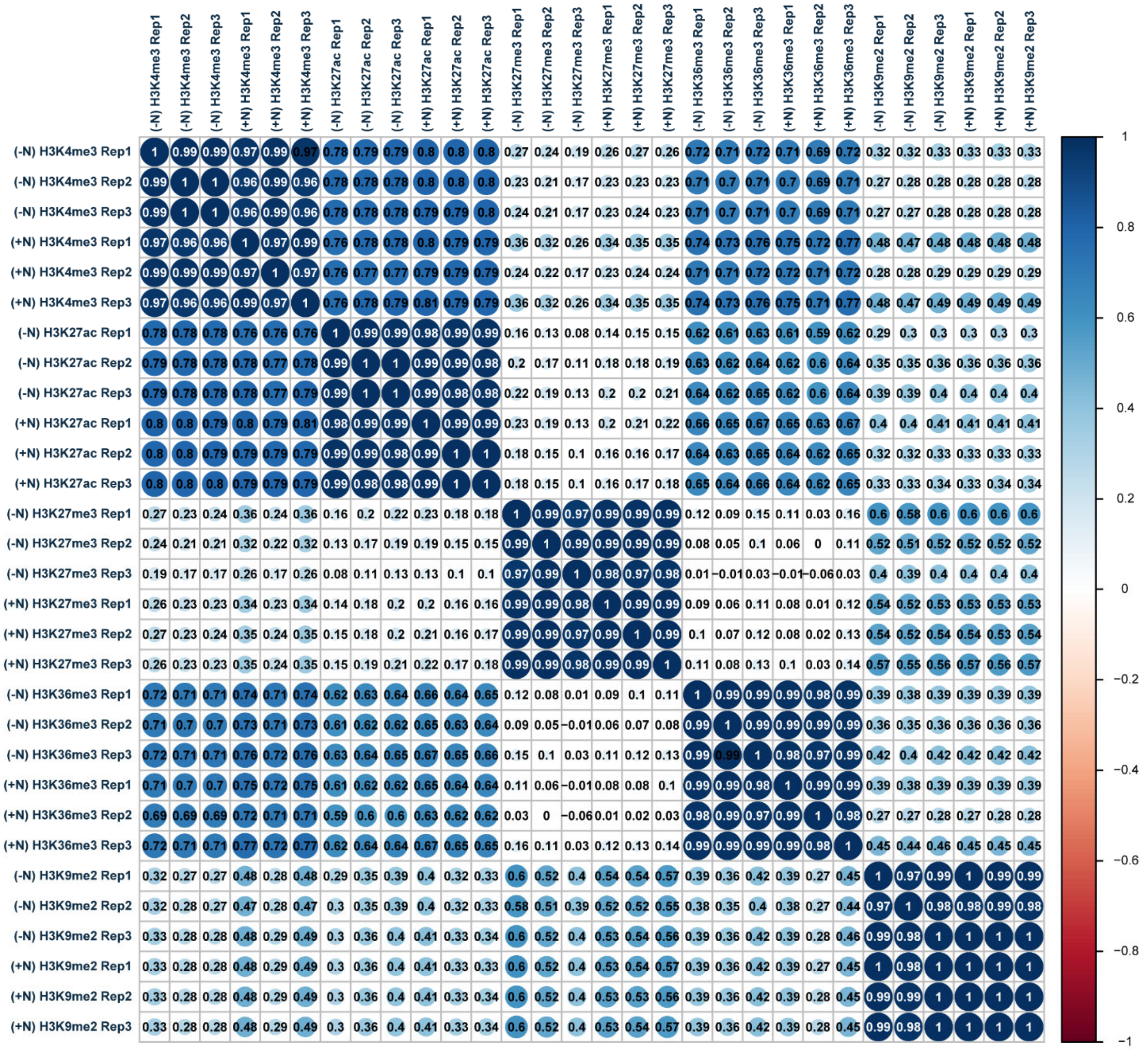

Supplementary Figure 7. Correlation between genic ChIP-Seq signal between replicates in shoot. Pearson correlation for ChIP-Seq signal (normalized by fragments per million and gene length, as used for input in machine learning predictions) for all annotated coding genes across all ChIP-Seq replicates in tomato shoot. Note that values ≥0.995 appear as 1.
