# Supplementary material for: Organ-specific characteristics govern the relationship between histone code dynamics and transcriptional reprogramming during nitrogen response in tomato
Source: Commun Biol. 2023 Dec 4;6:1225. doi: 10.1038/s42003-023-05601-8 (PMC10694154; doi:10.1038/s42003-023-05601-8)
Supplement: Supplementary file 2 — Description of Additional Supplementary Data [file 42003_2023_5601_MOESM2_ESM.docx]

**Description of Additional Supplementary Files**

**File name:** Supplementary Data 1

**Description:** Genes with significantly different histone modifications (DMGs) under N-supplied condition compared to N-starved controls in shoots or roots.

**File name:** Supplementary Data 2

**Description:** Differentially expressed genes (DEGs) in response to supply of nitrate in shoots and roots.

**File name:** Supplementary Data 3

**Description:** Significantly enriched GO terms among DEGs up-regulated or down-regulated in shoots or roots, determined by ShinyGO.

**File name:** Supplementary Data 4

**Description:** Significantly enriched GO terms among the DMGs with increased H3K27me3 at genic region and up-regulated in response to N in shoots.

**File name:** Supplementary Data 5

**Description:** Significantly enriched GO terms among top 10% DEGs predicted using H3K4me3 or H3K36me3 input to machine learning.

**File name:** Supplementary Data 6

**Description:** Genes encoding epigenetic regulators that are up- or down-regulated by N supply in shoots or in roots.

**File name:** Supplementary Data 7

**Description:** Significant GO terms enriched among DMGs.

**File name:** Supplementary Data 8

**Description:** Genes with significantly different H3K9me2 DMGs under N-supplied condition compared to N-starved controls in shoots or roots.

**File name:** Supplementary Data 9

**Description:** Source data for graphs in figures.
