# Supplementary material for: Organ-specific characteristics govern the relationship between histone code dynamics and transcriptional reprogramming during nitrogen response in tomato
Source: Commun Biol. 2023 Dec 4;6:1225. doi: 10.1038/s42003-023-05601-8 (PMC10694154; doi:10.1038/s42003-023-05601-8)
Supplement: Supplementary file 12 — Reporting Summary [file 42003_2023_5601_MOESM12_ESM.pdf]

nature portfolio

Corresponding author(s): Ying Li

Last updated by author(s): Oct 10, 2023

Reporting Summary

Nature Portfolio wishes to improve the reproducibility of the work that we publish. This form provides structure for consistency and transparency in reporting. For further information on Nature Portfolio policies, see our Editorial Policies and the Editorial Policy Checklist.

Statistics

For all statistical analyses, confirm that the following items are present in the figure legend, table legend, main text, or Methods section.

n/a	Confirmed	
☐	☒	The exact sample size (*n*) for each experimental group/condition, given as a discrete number and unit of measurement
☐	☒	A statement on whether measurements were taken from distinct samples or whether the same sample was measured repeatedly
☐	☒	The statistical test(s) used AND whether they are one- or two-sided<br>*Only common tests should be described solely by name; describe more complex techniques in the Methods section.*
☒	☐	A description of all covariates tested
☐	☒	A description of any assumptions or corrections, such as tests of normality and adjustment for multiple comparisons
☐	☒	A full description of the statistical parameters including central tendency (e.g. means) or other basic estimates (e.g. regression coefficient) AND variation (e.g. standard deviation) or associated estimates of uncertainty (e.g. confidence intervals)
☐	☒	For null hypothesis testing, the test statistic (e.g. *F*, *t*, *r*) with confidence intervals, effect sizes, degrees of freedom and *P* value noted<br>*Give P values as exact values whenever suitable.*
☒	☐	For Bayesian analysis, information on the choice of priors and Markov chain Monte Carlo settings
☒	☐	For hierarchical and complex designs, identification of the appropriate level for tests and full reporting of outcomes
☒	☐	Estimates of effect sizes (e.g. Cohen's *d*, Pearson's *r*), indicating how they were calculated

Our web collection on statistics for biologists contains articles on many of the points above.

Software and code

Policy information about availability of computer code

Data collection	No software was used for data collection
Data analysis	Cutadapt (v2.5), Tophat2 (v2.1.1), DESeq2 (v1.24.0), Bowtie2 (v2.3.3.1), SICER (v1.1), BEDTools (v2.29.0), htseq (v0.7.0), SAMtools (v1.8), in-house python script for normalization of read coverage at genome features, in-house python script for GO Term enrichment testing with FDR correction, XGBoost (v1.4.1.1), DescTools (v0.99.43)

For manuscripts utilizing custom algorithms or software that are central to the research but not yet described in published literature, software must be made available to editors and reviewers. We strongly encourage code deposition in a community repository (e.g. GitHub). See the Nature Portfolio guidelines for submitting code & software for further information.

Data

Policy information about availability of data

All manuscripts must include a data availability statement. This statement should provide the following information, where applicable:

- Accession codes, unique identifiers, or web links for publicly available datasets
- A description of any restrictions on data availability
- For clinical datasets or third party data, please ensure that the statement adheres to our policy

All raw and processed sequencing data generated in this study have been submitted to the NCBI Gene Expression Omnibus (GEO; https://www.ncbi.nlm.nih.gov/geo/) under accession number GSE196887.

Human research participants

Policy information about studies involving human research participants and Sex and Gender in Research.

Reporting on sex and gender	No human research was conducted
Population characteristics	No human research was conducted
Recruitment	No human research was conducted
Ethics oversight	No human research was conducted

Note that full information on the approval of the study protocol must also be provided in the manuscript.

Field-specific reporting

Please select the one below that is the best fit for your research. If you are not sure, read the appropriate sections before making your selection.

☒ Life sciences ☐ Behavioural & social sciences ☐ Ecological, evolutionary & environmental sciences

For a reference copy of the document with all sections, see nature.com/documents/nr-reporting-summary-flat.pdf

Life sciences study design

All studies must disclose on these points even when the disclosure is negative.

Sample size	Sample size of materials for CHIP-Seq and RNA-Seq was pooled tissue from four individual plants, this sample size was chosen from previous experience as sufficient to prevent large variation between samples
Data exclusions	No data was excluded from analysis
Replication	CHIP-Seq and RNA-Seq were performed with three distinct biological replicates which were jointly analyzed. Genome-wide correlation of genic ChIP-seq signal between different replicates was assessed.
Randomization	Plants were randomly assigned to control (+N) or (-N) blocks during hydroponic transfer and positionally rotated during growth. The samples were grown concurrently on the same bench space to avoid environmental variation.
Blinding	Blinding was not possible in this study as knowledge of the sample conditions is necessary to perform the analysis

Reporting for specific materials, systems and methods

We require information from authors about some types of materials, experimental systems and methods used in many studies. Here, indicate whether each material, system or method listed is relevant to your study. If you are not sure if a list item applies to your research, read the appropriate section before selecting a response.

Materials & experimental systems

n/a	Involved in the study	
☐	☒	Antibodies
☒	☐	Eukaryotic cell lines
☒	☐	Palaeontology and archaeology
☒	☐	Animals and other organisms
☒	☐	Clinical data
☒	☐	Dual use research of concern

Methods

n/a	Involved in the study	
☐	☒	ChIP-seq
☒	☐	Flow cytometry
☒	☐	MRI-based neuroimaging

Antibodies

Antibodies used	H3K4me3 (Millipore Sigma 07473), H3K27ac (Millipore Sigma 07360), H3K27me3 (Millipore Sigma 07449), H3K36me3 (Abcam ab9050), H3K9me2 (Abcam ab1220)
Validation	antibodies were validated for use in ChIP by the manufacturer for H3K4me3, H3K27ac, and H3K36me3, H3K9me2, and for IP for H3K27me3. all have previously been used in plant systems for published high quality ChIP-Seq studies.

ChIP-seq

Data deposition

☒ Confirm that both raw and final processed data have been deposited in a public database such as GEO.

☒ Confirm that you have deposited or provided access to graph files (e.g. BED files) for the called peaks.

Data access links<br>*May remain private before publication.*	To review GEO accession GSE196887:<br>Go to https://www.ncbi.nlm.nih.gov/geo/query/acc.cgi?acc=GSE196887<br>Enter token cribcyamdpabbil into the box
Files in database submission	C01_1.fq.gz C01_2.fq.gz<br>C02_1.fq.gz C02_2.fq.gz<br>C03_1.fq.gz C03_2.fq.gz<br>C04_1.fq.gz C04_2.fq.gz<br>C05_1.fq.gz C05_2.fq.gz<br>C06_1.fq.gz C06_2.fq.gz<br>C07_1.fq.gz C07_2.fq.gz<br>C08_1.fq.gz C08_2.fq.gz<br>C09_1.fq.gz C09_2.fq.gz<br>C10_1.fq.gz C10_2.fq.gz<br>C11_1.fq.gz C11_2.fq.gz<br>C12_1.fq.gz C12_2.fq.gz<br>C13_1.fq.gz C13_2.fq.gz<br>C14_1.fq.gz C14_2.fq.gz<br>C15_1.fq.gz C15_2.fq.gz<br>C16_1.fq.gz C16_2.fq.gz<br>C17_1.fq.gz C17_2.fq.gz<br>C18_1.fq.gz C18_2.fq.gz<br>C19_1.fq.gz C19_2.fq.gz<br>C20_1.fq.gz C20_2.fq.gz<br>C21_1.fq.gz C21_2.fq.gz<br>C22_1.fq.gz C22_2.fq.gz<br>C23_1.fq.gz C23_2.fq.gz<br>C24_1.fq.gz C24_2.fq.gz<br>C25_1.fq.gz C25_2.fq.gz<br>C26_1.fq.gz C26_2.fq.gz<br>C27_1.fq.gz C27_2.fq.gz<br>C28_1.fq.gz C28_2.fq.gz<br>C29_1.fq.gz C29_2.fq.gz<br>C30_1.fq.gz C30_2.fq.gz<br>C31_1.fq.gz C31_2.fq.gz<br>C32_1.fq.gz C32_2.fq.gz<br>C33_1.fq.gz C33_2.fq.gz<br>C34_1.fq.gz C34_2.fq.gz<br>C35_1.fq.gz C35_2.fq.gz<br>C36_1.fq.gz C36_2.fq.gz<br>C37_1.fq.gz C37_2.fq.gz<br>C38_1.fq.gz C38_2.fq.gz<br>C39_1.fq.gz C39_2.fq.gz<br>C40_1.fq.gz C40_2.fq.gz<br>C41_1.fq.gz C41_2.fq.gz<br>C42_1.fq.gz C42_2.fq.gz<br>C43_1.fq.gz C43_2.fq.gz<br>C44_1.fq.gz C44_2.fq.gz<br>C45_1.fq.gz C45_2.fq.gz<br>C46_1.fq.gz C46_2.fq.gz<br>C47_1.fq.gz C47_2.fq.gz<br>C48_1.fq.gz C48_2.fq.gz<br>C49_1.fq.gz C49_2.fq.gz<br>C50_1.fq.gz C50_2.fq.gz<br>C51_1.fq.gz C51_2.fq.gz<br>C52_1.fq.gz C52_2.fq.gz<br>C53_1.fq.gz C53_2.fq.gz<br>C54_1.fq.gz C54_2.fq.gz<br>C55_1.fq.gz C55_2.fq.gz<br>C56_1.fq.gz C56_2.fq.gz<br>C57_1.fq.gz C57_2.fq.gz<br>C58_1.fq.gz C58_2.fq.gz<br>C59_1.fq.gz C59_2.fq.gz<br>C60_1.fq.gz C60_2.fq.gz<br>R01_1.fq.gz R01_2.fq.gz<br>R02_1.fq.gz R02_2.fq.gz

R03_1.fq.gz R03_2.fq.gz
R04_1.fq.gz R04_2.fq.gz
R05_1.fq.gz R05_2.fq.gz
R06_1.fq.gz R06_2.fq.gz
R07_1.fq.gz R07_2.fq.gz
R08_1.fq.gz R08_2.fq.gz
R09_1.fq.gz R09_2.fq.gz
R10_1.fq.gz R10_2.fq.gz
R11_1.fq.gz R11_2.fq.gz
R12_1.fq.gz R12_2.fq.gz
T01_1.fq.gz T01_2.fq.gz
T02_1.fq.gz T02_2.fq.gz
T03_1.fq.gz T03_2.fq.gz
T04_1.fq.gz T04_2.fq.gz
T05_1.fq.gz T05_2.fq.gz
T06_1.fq.gz T06_2.fq.gz
T07_1.fq.gz T07_2.fq.gz
T08_1.fq.gz T08_2.fq.gz
T09_1.fq.gz T09_2.fq.gz
T10_1.fq.gz T10_2.fq.gz
T11_1.fq.gz T11_2.fq.gz
T12_1.fq.gz T12_2.fq.gz
GSE196887_raw_counts.txt.gz
GSE196887_root_SICER_df.tar.gz
GSE196887_shoot_SICER_df.tar.gz
GSE196887_shoot_SICER_df_K9.tar.gz

Genome browser session
(e.g. UCSC)

Provide a link to an anonymized genome browser session for "Initial submission" and "Revised version" documents only, to enable peer review. Write "no longer applicable" for "Final submission" documents.

Methodology

Replicates

Three independent biological replicates for each tissue and condition

Sequencing depth

All sequencing was paired end 2x150bp reads.
[sample] [read pairs] [unique concordant mapped pairs]
root-N INPUT-1 40,726,832 25,333,516
root-N INPUT-2 41,037,402 27,033,950
root-N INPUT-3 42,349,215 28,428,197
root+N INPUT-1 37,296,583 23,511,742
root+N INPUT-2 35,295,544 23,137,625
root+N INPUT-3 41,331,059 24,474,435
root-N K4me3-1 39,616,752 27,146,428
root-N K4me3-2 42,386,071 23,208,593
root-N K4me3-3 75,551,287 49,063,176
root+N K4me3-1 40,452,819 25,050,308
root+N K4me3-2 41,220,018 27,428,541
root+N K4me3-3 37,414,862 25,075,897
root-N K27ac-1 34,247,685 22,256,577
root-N K27ac-2 38,358,237 25,372,040
root-N K27ac-3 53,234,968 33,384,273
root+N K27ac-1 48,002,019 31,798,795
root+N K27ac-2 35,588,599 24,599,762
root+N K27ac-3 40,699,443 27,696,892
root-N K27me3-1 47,549,878 12,817,481
root-N K27me3-2 49,594,143 15,145,137
root-N K27me3-3 47,527,634 19,124,659
root+N K27me3-1 52,204,065 22,538,199
root+N K27me3-2 45,073,349 22,671,531
root+N K27me3-3 39,290,524 18,441,465
root-N K36me3-1 62,529,938 35,654,487
root-N K36me3-2 42,286,549 25,304,285
root-N K36me3-3 44,887,547 28,783,022
root+N K36me3-1 40,942,690 26,107,570
root+N K36me3-2 33,849,097 19,781,733
root+N K36me3-3 34,916,204 20,643,360
shoot-N INPUT-1 33,157,169 26,116,487
shoot-N INPUT-2 36,945,356 29,326,412
shoot-N INPUT-3 35,368,097 27,807,229
shoot+N INPUT-1 41,707,315 32,815,588
shoot+N INPUT-2 37,977,234 28,558,250
shoot+N INPUT-3 46,559,502 36,338,505
shoot-N K4me3-1 32,758,192 25,085,664
shoot-N K4me3-2 48,199,669 35,164,153
shoot-N K4me3-3 36,810,825 28,630,260

shoot+N K4me3-1 35,542,805 26,876,197
shoot+N K4me3-2 36,227,384 27,937,978
shoot+N K4me3-3 37,165,829 28,100,955
shoot-N K27ac-1 43,830,887 30,575,311
shoot-N K27ac-2 36,267,217 24,384,385
shoot-N K27ac-3 40,275,418 27,922,767
shoot+N K27ac-1 34,547,391 24,820,416
shoot+N K27ac-2 41,152,573 30,439,443
shoot+N K27ac-3 46,700,030 32,854,377
shoot-N K27me3-1 33,559,976 26,337,264
shoot-N K27me3-2 37,680,987 27,970,871
shoot-N K27me3-3 35,754,049 27,844,668
shoot+N K27me3-1 36,333,975 28,025,110
shoot+N K27me3-2 40,372,440 30,752,874
shoot+N K27me3-3 40,761,982 31,262,594
shoot-N K36me3-1 35,126,029 27,529,200
shoot-N K36me3-2 41,961,379 33,559,414
shoot-N K36me3-3 32,710,937 25,848,260
shoot+N K36me3-1 38,524,595 30,278,024
shoot+N K36me3-2 39,474,776 31,772,212
shoot+N K36me3-3 46,539,714 36,651,443
shoot-N INPUT-4 31,963,532 11,000,998
shoot-N INPUT-5 33,905,357 14,248,223
shoot-N INPUT-6 33,420,422 19,302,850
shoot+N INPUT-4 32,941,875 17,277,013
shoot+N INPUT-5 29,734,019 12,335,668
shoot+N INPUT-6 28,786,293 10,998,991
shoot-N K9me2-4 34,257,248 21,131,927
shoot-N K9me2-5 41,508,405 15,363,862
shoot-N K9me2-6 34,613,805 16,837,490
shoot+N K9me2-4 36,453,698 20,047,934
shoot+N K9me2-5 29,506,286 14,285,624
shoot+N K9me2-6 33,353,501 16,099,272

Antibodies

H3K4me3 (Millipore Sigma 07473), H3K27ac (Millipore Sigma 07360), H3K27me3 (Millipore Sigma 07449), H3K36me3 (Abcam ab9050) ,H3K9me2 (Abcam ab1220)

Peak calling parameters

bowtie2 --threads 20 -x $[Sl_genome_4.00] -1 $[READ1.fq] -2 $[READ2.fq] -S $[OUTPUT.sam]
sh SICER/SICER-df.sh $[+N_ChIP_sample.bed] $[+N_input_sample.bed] $[-N_ChIP_sample.bed] $[-N_input_sample.bed] 200 200 0.01 0.05
except for H3K27me3, where the parameters were 200 600 0.01 0.05

Data quality

Data quality was assured by only using consensus peaks present in the majority (2/3) of replicates and SICER performance was visually checked for accuracy in IGV.

Software

Cutadapt (v2.5), Bowtie2 (v2.3.3.1), SICER (v1.1), BEDTools (v2.29.0), SAMtools (v1.8), in-house python script for normalization of read coverage at genome features, in-house python script for GO Term enrichment testing with FDR correction, XGBoost (v1.4.1.1), DescTools (v0.99.43)
